# Definitions and standardized endpoints for the use of drug-coated balloon in coronary artery disease: consensus document of the Drug Coated Balloon Academic Research Consortium

**DOI:** 10.1093/eurheartj/ehaf029

**Published:** 2025-04-24

**Authors:** Simone Fezzi, Bruno Scheller, Bernardo Cortese, Fernando Alfonso, Raban Jeger, Antonio Colombo, Michael Joner, Eun-Seok Shin, Franz X Kleber, Azeem Latib, Tuomas T Rissanen, Simon Eccleshall, Flavio Ribichini, Ling Tao, Bon-Kwon Koo, Alaide Chieffo, Junbo Ge, Juan F Granada, Hans-Peter Stoll, Christian Spaulding, Rafael Cavalcante, Alexandre Abizaid, Takashi Muramatsu, Konstantinos Dean Boudoulas, Ron Waksman, Roxana Mehran, Donald E Cutlip, Mitchell W Krucoff, Gregg W Stone, Scot Garg, Yoshinobu Onuma, Patrick W Serruys

**Affiliations:** Department of Cardiology, University of Galway, University Road, Galway H91 TK33, Ireland; Division of Cardiology, Department of Medicine, Verona University Hospital, Verona, Italy; Clinical and Experimental Interventional Cardiology, University of Saarland, Homburg/Saar, Germany; Fondazione Ricerca e Innovazione Cardiovascolare, Milan, Italy; DCB Academy, Milan, Italy; Harrington Heart & Vascular Institute, University Hospitals Cleveland Medical Center, Cleveland, OH, USA; Department of Cardiology, Hospital Universitario de La Princesa, Universidad Autónoma de Madrid. IIS-IP, CIBERCV, Madrid, Spain; Department of Cardiology, Triemli Hospital Zürich, Zürich, Switzerland; Department of Cardiology, University of Basel, Basel, Switzerland; Cardio Center, Humanitas Clinical and Research Hospital IRCCS, Rozzano, Milan, Italy; Department of Cardiology, German Heart Center Munich, Technical University of Munich, Munich, Germany; Department of Cardiology, Ulsan University Hospital, University of Ulsan College of Medicine, Ulsan, South Korea; Mitteldeutsches Herzzentrum, University Halle-Wittenberg, Halle, Germany; Department of Cardiology, Montefiore Medical Center, Bronx, NY, USA; Heart Center, Central Hospital of North Karelia, Siunsote, Joensuu, Finland; School of Medicine, Faculty of Health Sciences, University of Eastern Finland, Kuopio, Finland; Department of Cardiology, Norfolk and Norwich University Hospital, Norwich, UK; Division of Cardiology, Department of Medicine, Verona University Hospital, Verona, Italy; Department of Cardiology, Xijing Hospital, Fourth Military Medical University, No. 15 Changle West Road, Xi’an, China; Department of Internal Medicine and Cardiovascular Center, Seoul National University Hospital, Seoul National University of College of Medicine, Seoul, South Korea; Department of Medicine, Vita Salute San Raffaele University, Milan, Italy; Interventional Cardiology, IRCCS San Raffaele Scientific Institute, Milan, Italy; Zhongshan Hospital, Fudan University, Shanghai, China; Cardiovascular Research Foundation, Columbia University Medical Center, New York, NY, USA; Orchestra BioMed, Inc.; New Hope, PA, USA; Department of Cardiology, European Hospital Georges Pompidou, Assistance Publique Hôpitaux de Paris and INSERM, Paris, France; Boston Scientific, Marlborough, MA, USA; Instituto do Coracao, Hospital das Clinicas, Faculdade de Medicina, Universidade de Sao Paulo, Sao Paulo, Brazil; Department of Cardiology, Fujita Health University Hospital, Toyoake, Japan; Division of Cardiovascular Medicine, The Ohio State University, Columbus, OH, USA; Section of Interventional Cardiology, MedStar Washington Hospital Center, Washington, DC, USA; The Zena and Michael A. Wiener Cardiovascular Institute, Icahn School of Medicine at Mount Sinai, New York, NY, USA; Beth Israel Deaconess Medical Center and Harvard Medical School, Boston, MA, USA; Department of Medicine, Duke University School of Medicine, Durham, NC, USA; The Zena and Michael A. Wiener Cardiovascular Institute, Icahn School of Medicine at Mount Sinai, New York, NY, USA; Department of Cardiology, Royal Blackburn Hospital, Blackburn, UK; School of Medicine, University of Central Lancashire, Preston, UK; Department of Cardiology, University of Galway, University Road, Galway H91 TK33, Ireland; Department of Cardiology, University of Galway, University Road, Galway H91 TK33, Ireland

**Keywords:** Drug-coated balloon, Randomized clinical trial, Academic Research Consortium, Coronary artery disease

## Abstract

The Drug Coated Balloon Academic Research Consortium project originated from the lack of standardization and comparability between studies using drug-coated balloons in the treatment of obstructive coronary artery disease. This document is a collaborative effort between academic research organizations and percutaneous coronary intervention societies in Europe, the USA, and Asia. This consensus sought to standardize study designs and endpoints for clinical trials involving drug-coated balloons, including defining angiographic, intravascular, and non-invasive imaging methods for lesion assessment, alongside considerations for post-revascularization pharmaco-therapy. The concept of ‘blended therapy’, which advocates for combining device strategies, is also discussed. This paper delineates study types, endpoint definitions, follow-up protocols, and analytical approaches, aiming to provide consistency and guidance for interventional cardiologists and trialists.

## Graphical Abstract

**Graphical Abstract:**
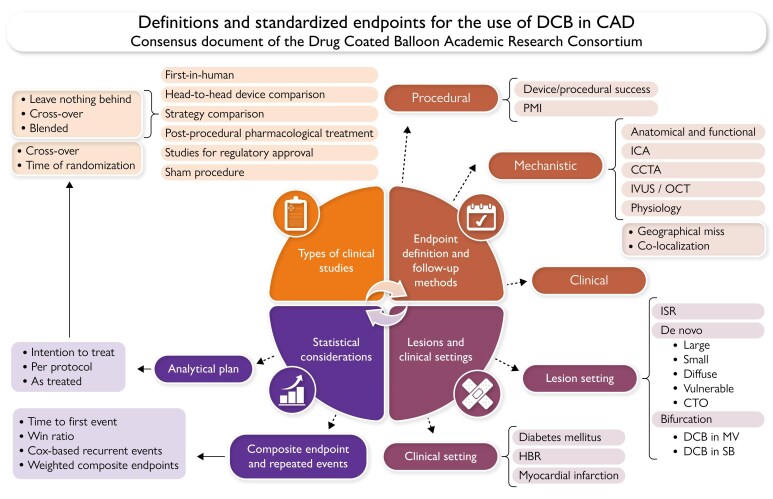
Definitions and standardized endpoints for the use of drug-coated balloon in coronary artery disease: consensus document of the Drug Coated Balloon Academic Research Consortium (DCB ARC). The DCB ARC initiative addresses the need for standardizing approaches in drug-coated balloon (DCB) research to enhance comparability between studies. This consensus aims to establish uniform study designs and endpoints for DCB clinical trials. Key components include standardized assessment parameters covering various imaging methods and post-revascularization therapy considerations. The paper outlines types of clinical studies, endpoint definitions and follow-up methods, lesions and clinical settings for the use of DCB, and statistical considerations, offering guidance and consistency to interventional cardiologists and trialists. CAD, coronary artery disease; CCTA, coronary computed tomography angiography; CTO, chronic total occlusion; DCB, drug-coated balloon; HBR, high bleeding risk; ICA, invasive coronary angiography; ISR, in-stent restenosis; IVUS, intravascular ultrasound; MV, main vessel; OCT, optical coherence tomography; PMI, peri-procedural myocardial infarction; SB, side branch

## Objectives

Drug-coated balloons (DCBs) are part of the armamentarium for the treatment of obstructive coronary artery disease (CAD). They have inherently different characteristics to drug-eluting stents (DES), relying on a fast and homogeneous transfer of antiproliferative drug into the vessel wall during balloon inflation, thereby removing the requirement for permanent vessel scaffolding and caging.^1,2^ Their use offers several distinct advantages over DES: (1) they ensure lesions remain amenable to regression with anti-atherogenic drugs; (2) they can be used in diffuse/small vessels/distal lesions where percutaneous coronary intervention (PCI) with stents yields sub-optimal results^3^; and (3) they ‘leave nothing behind’ preventing long-term permanent implant-related cardiovascular events.^4–6^ Nevertheless, the absence of metallic caging or radio-opaque markers that identify treated segments creates challenges (i.e. geographic miss) with analysing and co-localizing treated lesions at different time points and during final clinical event adjudication (i.e. restenosis, occlusion).

As part of the Academic Research Consortium (ARC), this document aims to standardize study designs for trials involving DCBs and define the recommended parameters for lesion assessment and trial endpoints, so that consistent, practical, and reproducible terminology is made available to interventional cardiologists and trialists in the field. These comprehensive definitions incorporate, among others, methods of angiographic assessment, as well as the role of intravascular imaging, non-invasive coronary imaging, new image-based methods of functional lesion evaluation, and dedicated post-revascularization anti-platelet therapy (type, duration, intensity) (*Graphical Abstract*).

Secondly, this document seeks to offer a high-level interpretation of the existing data in the field, which has been limited by the small sample sizes of clinical trials and poor or inconsistent-quality metrics. These challenges have contributed to the delay in producing large-scale, randomized clinical trials capable of impacting clinical practice guidelines.

Lastly, this document aims to define the emerging concept of ‘blended therapy’, namely the combination of various devices and technologies as a treatment strategy that can supersede the classical antagonism between various ‘*devices*’ of intervention.

Specifically, this position paper aims to describe:

1. Types of clinical studies performed with DCBs;
2. Endpoint definitions including composite clinical endpoints; procedural, mechanistic (anatomical and functional), and cost-effectiveness endpoints; patient-, site-, and central adjudication-reported endpoints;
3. Follow-up methods;
4. Analytical plans related to intention-to-treat, per-protocol, and as-treated analyses (with the option of sham treatment); the statistical approach for composite endpoints and interpretation of repeated events using various types of assessment;
5. Definitions of lesion and clinical settings for the use of DCB.

## Types of clinical studies in drug-coated balloon

The nomenclature commonly used in clinical trials investigating DCBs are described in *Table 1*, with the types of clinical studies using DCBs shown in *Table 2*. First-in-human, sham procedure and studies for regulatory approval are described in the Supplementary data.^7–13^

**Table 1:** Drug-coated balloon clinical trials’ nomenclature

	Nomenclature	Description
1	Drug-coated balloon (DCB)	Percutaneous coronary angioplasty balloon covered by antiproliferative drug, transferred homogenously into the vessel wall during a single balloon inflation by means of a carrier or a coating matrix
2	Drug-eluting balloon (DEB)^a^	Percutaneous coronary angioplasty balloon provided with delivery technologies (i.e. micro-pore) ensuring intraparietal drug release
3	Bail-out stenting/scaffolding	Implantation of a DES/BRS due to deterioration of flow (TIMI ≤2), flow-limiting dissections, or excessive recoil following pre-dilatation and/or DCB treatment, despite intracoronary medication (e.g. nitroglycerine, nitroprusside, calcium antagonist, nicorandil) are given and ∼5 min is waited
4	Cross-over	Change of intended pre-specified procedural strategy to another
5	Dissection Angiography	Mechanical disruption of the subintima, media, and/or adventitia layer of a coronary artery followed by extravasation of blood in the three above-mentioned layers, following lesion preparation and/or DCB treatment NHLBI classification (based on the depth and breadth of dissection and the presence of intimal flap or spiral appearance) Minor radiolucency within the lumen during contrast injection with no persistence after dye clearance Parallel tracts or double lumen separated by a radiolucent area during contrast injection with no persistence after dye clearance Extraluminal cap with persistence of contrast after dye clearance from the lumen Spiral luminal filling defects New persistent filling defect within the coronary lumen Non–A-E types that lead to impaired flow or total occlusion
	Intracoronary imaging	Tissue laceration categorized into intimal dissections, medial dissections, or adventitial dissections according to the depth the dissection reaches Morphometric measurements are: Cross-sectional: thickness, area, depth, aperture, and width (arc) of the dissected flap Longitudinal: length of the dissection Dissection volume: computation of the dissection area with the dissection length
6	Target lesion In-balloon In-segment	Lesion treated with DCB during the index procedure. Angiographic co-localization with DCB's markers or during DCB inflation is needed (i.e. co-registration, two different projections during DCB inflation, matched segment analysis using fiducial points). 1 mm proximal and distal to the balloon
7	Geographic miss	Angiographic mismatch between the lesion preparation (i.e. pre-dilatation with semi/non-compliant, cutting/scoring balloons, rotational or orbital atherectomy, IVL) and DCB application
8	Late lumen loss or gain	Difference between post-procedural and follow-up MLD
9	Acute recoil	Difference between balloon diameter and post-procedural MLD
10	Late recoil	Difference between balloon diameter and follow-up MLD
11	Acute gain	Difference between post- and pre-procedural MLD
12	Net gain	Difference between follow-up and pre-procedural MLD
13	Late functional loss/gain ΔFFR/QFR/iFR/FFR-CT	Paired difference of physiological epicardial values between post-procedure and follow-up. Fiducial co-localization is needed (PW sensor or distal marker of angiography-derived computation) Physiological drop across the targeted lesion (trans-DCB gradient, by analogy with trans-stent gradient), defined as the difference between instantaneous values assessed at the proximal and distal edges
14	Net functional gain	Paired difference of physiological epicardial values between pre-procedure and follow-up. Fiducial co-localization is needed (PW sensor or distal marker of angiography-derived computation)
15	Acute functional gain	Paired difference of physiological epicardial values between pre-procedure and post-procedure. Fiducial co-localization is needed (PW sensor or distal marker of angiography-derived computation)

BRS, bioresorbable scaffold; CT, computed tomography; DES, drug-eluting stent; FFR, fractional flow reserve; iFR, instantaneous wave-free ratio; IVL, intravascular lithotripsy; min, minutes; MLD, minimal lumen diameter; NHLBI, national heart, lung and blood institute; PW, pressure wire; QFR, quantitative flow ratio; TIMI, Thrombolysis In Myocardial Infarction.

^a^No RCT data are available yet for DEB. Clinical trials are still at phase I.

**Table 2:** Types of drug-coated balloon clinical trials

Type of study	Description	Procedural endpoints	Mechanistic (imaging and functional) endpoints	Clinical endpoints
First-in-human studies	Assessment of a new device's use feasibility and safety, with strict data safety monitoring and stopping rules	Procedural success: Device success Free from event during index hospitalization (CV death, TLF, PMI, any stroke)	Acute endpoints: Residual stenosis (QCA, IVUS, OCT) Acute gain Dissection (type, extension, classification) Perforation Post-procedural invasive functional assessment and/or image-based FFR IVUS/OCT: expansion index, dissection (volume, extension, length, depth) Shear stress Late endpoints: Late lumen loss (using the same method and projection as per post-procedural assessment) comparison with performance index derived from historical data Acute and net gain Binary restenosis (using the same method and projection as per post-procedural assessment) Invasive (e.g. PW-, IVUS-, OCT-based) or image-based FFR assessment using classical criteria of flow-limiting lesions (FFR ≤ 0.80)	Objective performance criteria: Safety endpoint: All-cause death CV death Stroke Any MI Definite lesion thrombosis (2) Efficacy endpoint: Any coronary revascularization TVR TLR (3) Composite efficacy and safety: CV death, TV-MI, and TLR (DOCE) All-cause death, any MI, and any revascularization (POCE) TV-MI, TLR, definite lesion thrombosis (LOCE)
Studies for regulatory approval	Assessment of device safety to obtain a CE Mark or Investigational device exemption (IDE/FDA/PMDA/CFDA). Non-inferiority comparison with available approved standard of care	Procedural success: Device success Free from event during index hospitalization (CV death, TLF, PMI, any stroke) Health–economic endpoints: Procedural time (min) Procedural cost Fluoroscopy time (min) Contrast medium amount (mL)	Acute endpoints: Residual stenosis (QCA, IVUS, OCT) Acute gain Dissection (type, extension, classification) Perforation Late endpoints: Late lumen loss or gain (using the same method and projection as per post-procedural assessment) Binary restenosis (using the same method and projection as per post-procedural assessment)	Short term (30 days) LOCE: Definite lesion thrombosis TLR (clinically driven) Device failure-related MI (not clearly attributable to a non-target vessel) Efficacy endpoint: TVR Target lesion-related ischaemia TLR Safety endpoint: BARC 3–5 Definite lesion thrombosis Any stroke Any MI CV death All-cause death
Device-comparing studies	Comparison of different devices: DCB vs. DES DCB vs. DCB ‘molecules eluted’ (e.g. paclitaxel vs. sirolimus/biolimus); ‘coating’ (e.g. hydrophilic matrix vs. phospholipid based vs. sub-micrometre nanoparticles) DCB vs. DEB	Procedural success: Device success Free from event during index hospitalization (CV death, TLF, PMI, any stroke) Myocardial injury (delta in troponin pre- vs. post-) Health–economic endpoints: Procedural time (min) Procedural cost Fluoroscopy time (min) Contrast medium amount (mL)	Acute endpoints: Residual stenosis (QCA, IVUS, OCT) Acute gain Dissection (type, extension, classification) Perforation Post-procedural invasive functional assessment and/or image-based FFR IVUS/OCT: expansion index, dissection (volume, extension, length, depth) IMR pre- and post-DCB Shear stress Late endpoints: Late lumen loss or gain (using the same method and projection as per post-procedural assessment) Binary restenosis (using the same method and projection as per post-procedural assessment) Invasive (e.g. PW-, IVUS-, OCT-based) or image-based FFR assessment using classical criteria of flow-limiting lesions (FFR ≤ 0.80)	DOCE: CV death Device failure-related MI Device failure-related ischaemia TLR Efficacy endpoint: TVR Target lesion-related ischaemia TLR Safety endpoint: BARC 3–5 Definite lesion thrombosis Any stroke Any MI CV death All-cause death
Strategy-comparing studies	Leave-nothing-behind strategy (cross-over to stent implantation either after pre-dilatation or DCB's inflation is a strategy failure) Cross-over strategy (cross-over stent implantation is according to the intended strategy based on operator's decision) Blended strategy and use of the complete PCI armamentarium (pre-specified combination of different technologies; i.e. DCB, DES, DEB, atherectomy, IVL, cutting/scoring balloons)	Intended primary strategy success (e.g. cross-over rate: the placement of a stent, as part of a declared leave-nothing-behind strategy in the pre-procedural planning, is considered a strategy failure) Procedural success: Device success Free from event during the index hospitalization (CV death, TLR, PMI, any stroke, BARC 3–5 bleeding) Health–economic endpoints: Procedural time (min) Procedural cost Fluoroscopy time (min) Contrast medium amount (mL)	Acute endpoints: Residual stenosis (QCA, IVUS, OCT) Acute gain Dissection (type, extension, classification) Perforation Post-procedural invasive functional assessment and/or image-based FFR IVUS/OCT: expansion index, dissection (volume, extension, length, depth) IMR pre- and post-DCB Late endpoints: Late lumen loss or gain (using the same method and projection as per post-procedural assessment) Binary restenosis (using the same method and projection as per post-procedural assessment) Invasive (e.g. PW-, IVUS-, OCT-based) or image-based FFR assessment using classical criteria of flow-limiting lesions (FFR ≤ 0.80)	DOCE: CV death Device failure-related MI Device failure-related ischaemia TLR Efficacy endpoint: TVR Target lesion-related ischaemia TLR Safety endpoint: BARC 3–5 Definite lesion thrombosis Any stroke Any MI CV death All-cause death
Post-procedural pharmacological comparison	Comparison between different anti-platelet strategies after PCI DAPT vs. aspirin-free monotherapy DAPT vs. SAPT Short DAPT vs. long DAPT	Final strategy adopted (i.e. leave-nothing-behind vs. cross-over strategy) Procedural success: Device success Free from event during the index hospitalizations (CV death, TLR, PMI, any stroke, BARC 3–5 bleeding)	Acute endpoints: Residual stenosis (QCA, IVUS, OCT) Acute gain Dissection (type, extension, classification) Perforation Post-procedural invasive functional assessment and/or image-based FFR IVUS/OCT: expansion index, dissection (volume, extension, length, depth) IMR pre- and post-DCB Late endpoints: Late lumen loss or gain (using the same method and projection as per post-procedural assessment) Binary restenosis (using the same method and projection as per post-procedural assessment) Invasive (e.g. PW-, IVUS-, OCT-based) or image-based FFR assessment using classical criteria of flow-limiting lesions (FFR ≤ 0.80)	Bleeding endpoint: BARC 3–5 bleeding POCE: All-cause death Any stroke Any MI Any revascularization NACE Bleeding endpoint POCE Nonadherence classifications according to NARC PROMs (i.e. SAQ)
Sham procedure studies	Sham procedure should be similar in every respect to the treatment being investigated with the exception that the active ingredient or component is lacking	Procedural success: Free from event during the index hospitalization (CV death, TLR, PMI, any stroke, BARC 3–5 bleeding)	Acute endpoints: Residual stenosis (QCA, IVUS, OCT) Acute gain Dissection (type, extension, classification) Perforation Post-procedural invasive functional assessment and/or image-based FFR IVUS/OCT: expansion index, dissection (volume, extension, length, depth) IMR pre- and post-DCB Late endpoints: Late lumen loss or gain (using the same method and projection as per post-procedural assessment) Binary restenosis (using the same method and projection as per post-procedural assessment) Invasive (e.g. PW-, IVUS-, OCT-based) or image-based FFR assessment using classical criteria of flow-limiting lesions (FFR ≤ 0.80)	POCE: All-cause death Any stroke Any MI Any revascularization NACE Bleeding endpoint POCE Nonadherence classifications according to NARC PROMs (i.e. SAQ)

BARC, Bleeding Academic Research Consortium; CE, Conformité Européenne; CFDA, China food and drug administration; CV, cardiovascular; DAPT, dual anti-platelet therapy; DCB, drug-coated balloon; DEB, drug-eluting balloon; DES, drug-eluting stent; DOCE, device-oriented composite endpoint; FDA, food and drug administration; FFR, fractional flow reserve; IDE, investigational device exemption; IMR, index of microvascular resistance; IVL, intravascular lithotripsy; IVUS, intravascular ultrasound; LOCE, lesion-oriented composite endpoint; MI, myocardial infarction; min, minutes; mL, milliliters; NACE, net clinical benefit composite endpoint; NARC, non-adherence academic research consortium; OCT, optical coherence tomography; PCI, percutaneous coronary intervention; PMDA, pharmaceuticals and medical devices agency; PMI, peri-procedural myocardial infarction; POCE, patient-oriented composite endpoint; PROMs, patient-reported outcome measures; PW, pressure wire; QCA, quantitative coronary angiography; SAPT, single-anti-platelet therapy; SAQ, Seattle Angina Questionnaire; TLF, target lesion failure; TLR, target lesion revascularization; TV-MI, target vessel myocardial infarction; TVR, target vessel revascularization.

### Head-to-head device-comparing studies

These studies aim at comparing the performance of new devices using standard of care as reference or another promising innovative device. These comparisons can be inter-device (e.g. DCB vs. DES), such as the Balloon Elution and Late Loss Optimization trial (BELLO)^14^ or intra-device (e.g. DCB vs. DCB), which includes comparing DCBs delivering different antiproliferative drugs, e.g. the TRANSFORM I study.^15^ The focus of these studies is primarily procedural success and efficacy, which is best fulfilled using procedural, imaging and functional endpoints.

### Strategy-comparing studies

This category includes studies comparing different strategies and philosophies of using DCBs in the management of CAD. Pre-procedure it should be clear whether the study is a strict comparison between treatments or whether a ‘blended treatment approach’ is permitted. In the former, failure of the DCB strategy and cross-over to the comparator group is considered a strategy failure inducing a penalty for the composite endpoint, while in the latter, a mixture of the two technologies is allowed by protocol to a pre-defined limit, and the blending of devices is not considered a failed treatment strategy.

In both types of study, the intended strategy must be declared and characterized pre-procedure, with the study protocol clearly describing the procedural and clinical scenarios allowed within each. Typically, the randomization should indicate the first steps of the strategy, with the subsequent stages documented according to the previous responses (e.g. cross-over to stent after initial pre-dilatation). If the strategy allows multiple procedural scenarios, details of the procedure should be recorded.

For example, according to the ‘leave nothing behind’ strategy, the intended primary success is achieved when a DCB treatment is carried out without cross-over interventions, which would be considered a strategy failure. In contrast, when the primary comparison is between a DCB and DES, and the DCB strategy allows cross-over to implantation of a DES (the ‘cross-over’ strategy), this is not considered a strategy failure. This second option examines the real clinical value of the technology in different clinical or anatomic settings. The SELUTION DeNovo trial compares in terms of target vessel failure a strategy of PCI with provisional DCB and rescue DES vs. intended DES implantation. Non-inferiority is tested at 1 and 5 years, and if met at 5 years, superiority will be tested.^16^

The REC-CAGEFREE I trial demonstrated that in patients with *de novo*, non-complex lesions, a strategy of provisional DCB angioplasty with rescue stenting did not achieve non-inferiority compared with intended DES implantation in terms of the occurrence of the device-oriented composite endpoint at 2 years. However, a pre-defined and powered analysis of vessel sizes, particularly those smaller than 3 mm (which represent 48% of the studied population), demonstrated that DCB was non-inferior to DES in vessels smaller than 3 mm.^17^

Lastly, according to the ‘blended’ strategy, the pre-specified use of the complete available armamentarium (i.e. DCB, DES, bioresorbable scaffolds [BRS], intravascular lithotripsy [IVL], scoring/cutting balloons) is allowed. The Drug Coated Balloon Academic Research Consortium (DCB ARC) highlights the potential of using such strategies in several settings, such as treatment of diffuse disease or multivessel disease. In this subgroup, the mixture of available technologies is part of the strategy (e.g. calcium debulking technologies comparison) which finishes with using a DCB, while the comparator could be the use of a DES or even surgical revascularization.

The time of randomization is a crucial factor that is influenced by the type of study and must be pre-defined according to the study protocol: upfront randomization occurring before lesion preparation and DCB treatment is preferable in studies comparing strategies. Conversely, in studies comparing devices, the investigated treatment should not be influenced by the result of lesion preparation, and therefore randomization should occur once treatment with a DCB strategy is felt to be suitable, with this approach allowing alignment between the two cohorts.

### Post-procedural pharmacological comparison

The international DCB consensus recommendation for 4 weeks of dual anti-platelet therapy (DAPT) following DCB treatment in *de novo* chronic coronary syndrome is based on expert opinion and the promising results from recent clinical trials.^18,19^ DCBs however have the potential to facilitate early P2Y_12_ de-escalation or discontinuation of DAPT (P2Y_12_ or aspirin discontinuation), which is particularly attractive in the high bleeding risk (HBR) population.^20^ In a recent all-comers real-world registry, which included HBR patients (65% on oral anticoagulation), DCB treatment followed by a single anti-platelet regimen was shown to be safe.^21^ To date, however, no outcomes studies are available testing the use of P2Y_12_ inhibitor monotherapy (aspirin-free strategy)^22,23^ or different anti-platelet regimens after DCB treatment. The latter encompasses numerous permutations such as comparing different P2Y_12_ inhibitors (clopidogrel vs. prasugrel vs. ticagrelor), DAPT vs. single-anti-platelet therapy (SAPT; aspirin or aspirin-free), reduced DAPT duration, or early de-escalation from a more potent agent to clopidogrel. Ultimately, evaluating these scenarios in dedicated trials will help establish the optimal DAPT strategy after DCB/DES procedures.

## Endpoint definitions

According to DCB ARC, endpoints for clinical studies can be categorized as *procedural*, *mechanistic* (anatomical and functional), and *clinical*.

*Procedural* endpoints encompass procedure-related outcomes and are relevant for all types of clinical study. The definition of procedural success includes the concept of device success, freedom from adverse events during the index hospitalization [cardiovascular death, target lesion failure, peri-procedural myocardial infarction (PMI), and stroke] and peri-procedural myocardial injury, which might be of greatest interest in studies comparing devices. In studies comparing strategies, procedural endpoints should include the ‘intended primary strategy success' that nevertheless permits cross-over from the planned strategy.

*Mechanistic* endpoints include imaging and functional efficacy endpoints derived from invasive (angiography, intracoronary imaging, invasive coronary physiology) and non-invasive [coronary computed tomography angiography (CCTA), fractional flow reserve derived from computed tomography (FFR_CT_)] assessments. They are intended to report the mechanical result of the procedure being investigated and generally include a pre-procedure, post-procedure, and follow-up assessment and should be assessed by an independent and blinded core lab using standardized methodology.

*Clinical* endpoints include the occurrence of individual and composite safety and efficacy endpoints. The choice of composite endpoints, which can include device-, lesion-, patient-, and net adverse clinical event-related endpoints, is based on the type of clinical study being performed.

The DCB ARC proposes specific individual and composite endpoints according to the type of clinical study being considered. Potential endpoints should be blindly adjudicated by an independent Clinical Events Committee (CEC) based on redacted source documents and supported by core lab evaluation.

### Trials aimed at device/procedural success

The primary aim of first-in-human studies is to test the feasibility (device success) and safety (early safety) of the device, and since these studies usually have limited statistical power, pre-specified performance goals are often used as criteria for success or failure [e.g. ASET (Acetyl Salicylic Elimination Trial) pilot study^24^]. An independent data safety monitoring board is mandatory, with their role advising continuation or discontinuation of the trial with respect to safety concerns (pre-defined stopping rules). Subsequently, clinical registries and small randomized controlled trials (RCTs) can be undertaken aimed at investigating the clinical efficacy of the technology, taking advantage of the comparison with pre-existing objective performance criteria,^7^ and using performance indexes stemming from well-established historical data.

In trials comparing devices and/or strategies, procedural and mechanistic (imaging and functional) endpoints are of particular importance: imaging and functional endpoints are based on post-procedural and mid-term follow-up assessment (cf. Follow-up methods). Preferentially, such analyses should be performed by an independent and blinded core laboratory having standardized operational methodology and pre-defined analytical plans. The DCB ARC recommendation for angiography, physiology, and intravascular imaging analysis included herein should be strictly followed. If a core lab analysis is planned, a test run should be performed to assess the adequacy of data acquisition prior to starting the trial. In this specific subgroup of trials, clinical endpoints play a secondary role and should be set as secondary endpoints.

### Trials investigating clinical benefit

The primary outcome measures in studies for regulatory approval should be clinical endpoints of safety and performance (efficacy), with surrogate endpoints ancillary. Adequately powered device- and strategy-comparing trials should aim at comparing clinical benefit of the investigated device/strategy.

The primary outcomes in trials comparing post-procedural pharmacological and in sham procedure studies should be clinical composite endpoints with safety (e.g. bleeding events), ischaemic, and patient-oriented composites of greatest relevance. Net adverse clinical events that incorporate safety-related and patient-reported outcomes should also be reported. In sham procedure studies, on top of patient-oriented endpoints, patient-reported outcome measures (PROMs) play a key role, with the comparison between the two study arms potentially having a significant impact on the patient's perceived health status.

### Individual endpoints

#### Procedural endpoint

The international DCB consensus^1^ proposes that, in clinical practice, an optimal balloon angioplasty comprises (i) a fully inflated balloon of the correct size for the vessel; (ii) ≤30% residual stenosis by visual estimation; (iii) Thrombolysis In Myocardial Infarction (TIMI) flow grade 3; and (iv) the absence of flow-limiting dissections. However, visual estimation in the assessment of post-angioplasty residual stenosis is flawed by significant investigator-dependent variations of ≥10%^25^; therefore, in clinical trials, the use of quantitative coronary analysis (QCA) is recommended. Notably, QCA evaluation after DCB PCI can be hampered by dissections and cannot detect and depict accurately complex intra-luminal dissections, not visible on the luminal contours. The need for the development of dedicated QCA protocols might emerge in future.

In clinical trials, device success is defined as the composite of successful delivery within a reasonable transfer time to the target lesion (e.g. <2 min) and inflation for 30–60 s of the allocated DCB device at the intended target lesion during an attempt with a DCB not previously used (first use), with successful withdrawal of the device system, while attaining a final in-segment or in-lesion residual per cent diameter stenosis (%DS) of <40% by off-line core lab adjudicated QCA.^26^

According to the CAGEFREE (NCT04561739) real-world registry of 2473 patients treated for *de novo* and in-stent restenosis (ISR) lesions, the median %DS after DCB treatment was 30%, with a %DS <20% and <50% achieved in 20% and >90%, respectively.^27^ Therefore, DCB ARC recommends that device success after DCB treatment should be considered when the %DS < 40% is achieved using off-line QCA; however, a post-procedural stratification into optimal (%DS < 30%) and sub-optimal (30% < %DS < 40%) is also supported. When DCBs are being directly compared to DES, DCB ARC recommends using specific thresholds for each device (<40% for DCB, <20% for DES).

Stent implantation may be performed for sub-optimal results after lesion preparation (i.e. a dissection or unacceptable recoil) or, if necessary, as bail-out after DCB application.

In contrast to PCI using coronary stents, no validated cut-offs for procedural success using intravascular imaging have yet been validated for DCBs. Depending on the study design, the use of bail-out devices (as allocated by randomization) due to severe dissections (type C-F) or impaired coronary flow (TIMI <3) may or may not be judged as device failure.

Procedural success herein is ascertained at discharge as the composite of device success plus the absence of adverse procedural clinical outcomes including cardiovascular death, target lesion revascularization, PMI, any stroke, and Bleeding Academic Research Consortium (BARC) 3 or 5 bleeding, regardless of whether the protocol-assigned device is used. In the ‘leave-nothing-behind’ strategy trials, a penalty for cross-over is included in the composite assessment of procedural success.

#### Clinical endpoints

Individual endpoint definitions are reported in *Table 3*, composite endpoints in *Table 4*, and surrogate endpoints in *Table 5*.

**Table 3:** Individual endpoints’ definition

	Nomenclature	Description
1	Device success	All of: Successful delivery in time and inflation within 30–60 s of the allocated DCB device at the intended target lesion during an attempt with a DCB not previously used (first use) Successful withdrawal of the device system Attainment of a final in-segment or in-lesion residual stenosis of <40% with final data reported by core laboratory QCA (preferred methodology)
2	Procedure success	All of: Device success Freedom from in-hospital cardiovascular death, target lesion revascularization, peri-procedural myocardial infarction, any stroke, and BARC 3–5 bleeding Freedom from bail-out stenting (for ‘leave-nothing-behind’ strategy)
3	Cardiovascular death	Death caused by acute MI Sudden cardiac, including unwitnessed, death Death resulting from heart failure Death caused by stroke Death caused by cardiovascular procedures Death resulting from cardiovascular haemorrhage (haemorrhage deriving from cardiac and/or vascular disease/injuries)
4	Peri-procedural MI	Evaluation 24–48 h: hs-cTn T rise ≥35xURL AND ≥1 of the following criteria: ‘Flow-limiting’ angiographic complications in a major epicardial vessel at the end of the procedure New significant Q-waves (or equivalent) in two contiguous leads, after the procedure A new wall motion abnormality on echocardiography, after the procedure OR hs-cTn T rise ≥70xURL (all events should be adjudicated, ideally after core lab analysis, by an independent CEC)
5	Cardiac biomarker rise	Any CK-MB and/or hs-cTn T rise >6 h after the procedure Type 1: due to other angiographic complications Intraprocedural occlusion of the target vessel Intraprocedural distal embolization Intraprocedural coronary perforation Intraprocedural dissection (after pre-dilatation, after DCB) Residual flow-limiting dissection at the end of the procedure Intraprocedural lesion thrombus Residual thrombus at the end of the procedure Increased IMR or angio-IMR (≥25) at the end of the procedure Type 2: no angiographic identifiable causes
6	Stroke	Neuro-ARC definitions (according to ARC-2 criteria)
7	Bleeding	BARC definitions (according to ARC-2 criteria)
8	Target lesion ischaemia	The target lesion ischaemia is defined in presence of ischaemic myocardium supplied by the coronary segments treated during the initial procedure. Identification and localization of ischaemia requires the use of the same ischaemic test, utilized during the inclusion in the study
9	Target lesion revascularization	The target lesion is considered as the treated coronary segment during the index procedure plus 1 mm distance from the balloon edges Target lesion revascularization is defined as a repeat percutaneous intervention of the target lesion or bypass surgery of the target vessel performed for restenosis or other complication of the target lesion
10	Target vessel revascularization	The target vessel is defined as the entire major treated coronary vessel, including side branches Target vessel revascularization is defined as any repeat percutaneous intervention or surgical bypass of any segment of the target vessel
11	Target lesion-related MI	Any MI associated with angiographic confirmation that the culprit lesion corresponds to the DCB-treated segment (1 mm proximal and distal to the balloon)
12	Target vessel non-target lesion MI	Any MI attributed to the target vessel, but not involving the target lesion's segment
13	Definite lesion thrombosis	Angiographic confirmation: the presence of a thrombus that originates the segment 1 mm proximal or distal to the treated lesion and the presence of at least one of the following criteria: Acute onset of ischaemic symptoms at rest New electrocardiographic changes suggestive of acute ischaemia Typical rise and fall in cardiac biomarkers (refer to definition of spontaneous myocardial infarction) OR Pathological confirmation: Evidence of recent thrombus within the target lesion determined at autopsy Examination of tissue retrieved following thrombectomy (visual/histology) Early acute: 0–24 h; early subacute: 1–30 days; late: 30 days–1 year; very late: >1 year
14	Probable lesion thrombosis	Regardless of the time after the index procedure, any myocardial infarction that is related to documented acute ischaemia in the territory of the treated lesion without angiographic confirmation of thrombosis and in the absence of any other obvious cause Early acute: 0–24 h; early subacute: 1–30 days; late: 30 days–1 year; very late: >1 year
15	Silent target segment occlusion	The incidental angiographic documentation of DCB-treated segment occlusion in the absence of clinical signs or symptoms. Silent target segment occlusion is not adjudicated as lesion thrombosis.
16	Major dissection	Dissection in the target lesion ≥ type C the from National Heart, Lung, and Blood Institute classification
17	Perforation	Type 1) extraluminal crater without jet extravasation Type 2) pericardial or myocardial blushing without jet extravasation Type 3) active jet extravasation exit jet >1 mm Type 4) leaking into another cardiovascular cavity Type 5) distal perforation
18	Binary stenosis	>50%DS in the target segment at follow-up

ARC, Academic Research Consortium; BARC, Bleeding ARC; CEC, Clinical Events Committee; CK-MB, creatine kinase MB; d, day; DCB, drug-coated balloon; DS, degree of stenosis; hs-cTnT, high-sensitivity troponin T; IMR, index of microvascular resistance; MI, myocardial infarction; mm, millimiter; QCA, quantitative coronary analysis; URL, upper reference limit; y, years.

**Table 4:** Composite endpoints’ definition

	Nomenclature	Description
1	DOCE	Hierarchical occurrence of: Cardiovascular mortality Device failure-related MI (not clearly attributable to a non-target vessel) Device failure-related ischaemia TLR (clinically driven)
2	VOCE	Hierarchical occurrence of: Vessel-related cardiac death Target vessel MI (not clearly attributable to a non-target vessel) TVR
3	POCE	Hierarchical occurrence of: All-cause mortality Any stroke Any MI (includes non-target vessel territory) Any revascularization
4	LOCE	Hierarchical occurrence of: Definite lesion thrombosis TLR (clinically driven) Device failure-related MI (not clearly attributable to a non-target vessel)
5	Functional LOCE	Hierarchical occurrence of: Definite lesion thrombosis TLR (clinically driven) Device failure-related MI (not clearly attributable to a non-target vessel) Trans-DCB functional gradient ≥ 0.06
6	MACE	Hierarchical occurrence of: All-cause mortality Any MI Any stroke Hospitalization for heart failure Any revascularization
7	NACE	POCE Bleeding type 3 or 5 according to the Bleeding ARC
8	TVF	Cardiovascular death Target vessel MI TVR
9	TLF	Cardiovascular death Target vessel MI TLR (clinically driven)
10	Safety endpoints	Bleeding type 3 or 5 according to the Bleeding ARC Definite lesion thrombosis Any stroke Any MI Cardiovascular mortality All-cause mortality
11	Efficacy endpoints	Target vessel revascularization Device failure-related ischaemia TLR

ARC, academic research consortium; DCB, drug coated balloon; DOCE, device-oriented composite endpoint; LOCE, lesion-oriented composite endpoint; MACE, major adverse cardiac event; MI, myocardial infarction; NACE, net adverse clinical events; POCE, patient-oriented composite endpoint; TLF, target lesion failure; TLR, target lesion revascularization; TVF, target vessel failure; TVR, target vessel revascularization; VOCE, vessel-oriented composite endpoint.

**Table 5:** Surrogate endpoints for drug-coated balloon clinical trials

Endpoints		Advantages	Disadvantages
Coronary computed tomography angiography			
Minimal lumen area (MLA)	Minimal lumen area along the length of the target lesion	Non-invasive assessment No drawbacks in case of calcified or tortuous vessel Definition of the amount of plaque, remodelling, functional assessment PcD eliminate blooming artefacts associated with calcium or metallic struts while improving the delineation of low-attenuation areas	Complexity of assessing the geographical miss on CCTA No metal present (fiducial co-localization with side branches) Small vessels (1.5 mm) beyond the temporal resolution of CCTA Blooming artefact due to severe calcification (not for PcD)
Plaque burden (PB)	Plaque area divided by the cross-sectional area of the EEM		
Remodelling	Outer vessel diameter of the lesion divided by the outer vessel diameter of the reference normal segment in the same vessel		
Vessel patency			
FFR-CT	Distal vessel FFR-CT, ΔFFR-CT across the treated segment		
Coronary angiography			
Acute gain	Difference between post- and pre-procedural MLD	Gold standard No need for dedicated PW or imaging catheters Costs Possibility of co-localization with the DCB-treated segment Possibility of angiography-derived physiology computation	Low spatial resolution Limited assessment of dissections Limited assessment of thrombus No assessment of plaque composition and morphology
Net gain	Difference between follow-up and pre-procedural MLD		
Late lumen loss or gain	Difference between post-procedural and follow-up MLD		
Degree of stenosis change	Difference between post-procedural and follow-up %DS		
Intracoronary imaging (IVUS, OCT)			
Minimal lumen area (MLA)	Minimal lumen area along the length of the target lesion	Evaluation of dissections (dissection classification/quantitative assessment) Plaque composition and morphology Thrombus presence exclusion Pre-dilatation result and DCB sizing Angio-imaging co-registration and accurate longitudinal measurement Functional assessment with OFR or UFR	Lower resolution with IVUS (difficult to assess and classify dissections) Need for high pressure contrast injection with OCT (potential worsening of dissections) Cost of the device Attrition Potential bias for the event case adjudication
Plaque burden (PB)	Plaque area divided by the cross-sectional area of the EEM		
Neointimal area (mean/max) and volume	Difference between stent and minimal lumen area and computation of the neointimal area with the lesion length		
Remodelling	CSA of the lesion EEM divided by the CSA of the reference EEM		
Dissection volume/extension	Computation of the dissection area with the longitudinal dissection length		
Expansion index	MLA divided by the average reference lumen area		
Coronary physiology (FFR, iFR, QFR)			
Vessel FFR/QFR	Functional pressure drop along the entire vessel	Assessment of the physiological relevance of a given stenosis Assessment of physiological pattern of coronary disease Assessment of microcirculation Angiographic co-registration and co-localization with the treated segment (QFR and iFR Syncvision)	Need for dedicated PW and for hyperaemic agents (in case of FFR and PPGi)
Trans-DCB gradient (TDCBG)	Trans-segment pressure gradient measured by iFR PW pullback co-registration (Syncvision) or by the instantaneous QFR value on the virtual pullback		
PPGi–QVPi	Magnitude of pressure drop over 20 mm and the extent of functional disease in order to assess the functional pattern of disease (focal vs. diffuse)		
dFFR/dT–dQFR/dS	Local functional disease severity		
IMR–angio-IMR	Microvascular resistance and coronary microvascular function		

CCTA, coronary computed tomography angiography; CSA, cross-sectional area; CT, computed tomography; DCB, drug-coated balloon; DS, degree of stenosis; EEM, external elastic membrane; FFR, fractional flow reserve; iFR, instantaneous wave-free ratio; IMR, index of microvascular resistance; IVUS, intravascular ultrasound; MLA, minimal lumen area; MLD, minimal lumen diameter; OCT, optical coherence tomography; OFR, optical flow ratio; PcD, photon-counting detector CT; PPGi, pullback pressure gradient index; PW, pressure wire; QFR, quantitative flow ratio; QVPi, QFR virtual pullback index; dT, unit time; dS, unit space; UFR, ultrasonic flow ratio.

### Myocardial infarction

#### Peri-procedural myocardial infarction

Myocardial infarctions (MIs) may occur in the peri-procedural phase, as well as during follow-up, either due to a spontaneous event or late complications related to the investigated device/strategy. Several definitions of MI, and in particular PMI, have been proposed by different cardiac societies and adopted in different clinical trials.^28,29^ Specific criteria should be adopted to define the occurrence and the clinical relevance of MIs, according to study type and design, in order to properly weigh the sensitivity of cardiac biomarkers of subtle myocardial injury (e.g. troponin I, troponin T) and balance them against clinically relevant adverse events.^28^

Notably, while contemporary definitions of PMI largely rely on high-sensitivity cardiac troponin (hs-cTn), no correlation between the different types of available hs-cTn assays has been clearly established, hampering the comparisons between different studies.

The DCB ARC supports the Society for Cardiovascular Angiography and Interventions (SCAI) PMI definition, by using hs-cTn T, measured with a single assay within 24–48 h of the PCI. In DCB studies, a PMI is defined as an absolute increase in hs-cTnT ≥35 × upper limit of normal (ULN) combined with clinical evidence of MI, or as an absolute increase of hs-cTnT ≥70 × ULN.^30^

Due to the complexity and uniqueness of the definition and adjudication of PMI, and since a sensitive and inclusive definition of PMI could potentially drive most of the composite clinical endpoints and, even if equally affecting the two arms, may drastically affect the study results, ARC is simultaneously working on a document dedicated to the definition of PMI. DCB ARC will be updated accordingly.

Post-procedural cardiac biomarkers rise, and spontaneous MI definitions are reported in the Supplementary data.^31–33^

### Bleeding

Bleeding events should be classified and reported according to the BARC criteria.^34^ Type, intensity, and duration of anti-platelet medication at the time of bleeding should be captured. Significant bleeding is categorized as BARC 3–5 bleeding.^35^ BARC 2 bleeding may also be included to enhance the power calculation for composite endpoints. It is important to note that these are nuisance events with limited clinical relevance, and their inclusion might reduce the sensitivity and specificity of bleeding assessment.

### Repeat revascularizations

Repeat revascularizations are defined according to the vessel/lesion treated and are identified as target or non-target, based on the initial site of the DCB treatment. The target lesion is considered as the treated coronary segment during the index procedure plus 1 mm from the proximal and distal edge of the DCB; accurate angiographic segment co-localization is needed (see Follow-up methods).

**Figure 1:**
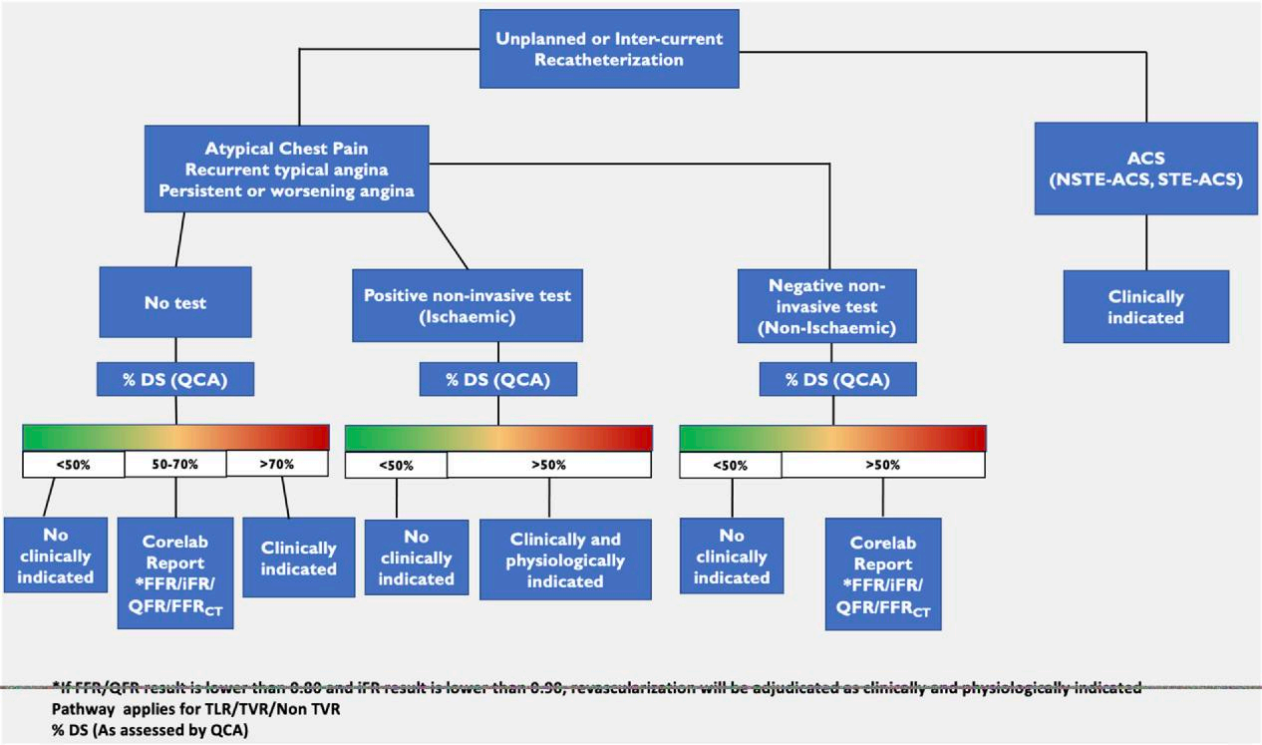
Process for inter-current core lab event adjudication during unplanned or follow-up catheterizations. On the right side of the panel, in cases of acute coronary syndromes, coronary angiography and intervention are considered clinically indicated and adjudicated as an event. For stable situations, such as atypical chest pain or typical recurrent/persistent/worsening angina, the evaluation of ischaemia through non-invasive tests is crucial. If non-invasive tests indicate ischaemia, and quantitative coronary analysis shows >50% stenosis in the target lesion, revascularization is deemed clinically and physiologically appropriate and adjudicated as an event. If quantitative coronary analysis shows 50%–70% stenosis, physiological assessment using pressure-derived (fractional flow reserve/instantaneous wave-free ratio) or angiography-derived methods (quantitative flow ratio/fractional flow reserve derived from computed tomography) is necessary to justify revascularization from a physiological and clinical perspective and to adjudicate it as an event. In cases of angiographic assessment which have not been preceded by non-invasive ischaemic diagnostic tests, which is not the preferred clinical approach, but required in some mechanistic studies with specified angiographic follow-ups, revascularization is considered clinically indicated if the stenosis exceeds 70% by quantitative coronary analysis or 90% by visual estimation and adjudicated as an event. For stenoses between 50% and 70% by quantitative coronary analysis, physiological assessment through pressure-derived (fractional flow reserve/instantaneous wave-free ratio) or angiography-derived methods (quantitative flow ratio/fractional flow reserve derived from computed tomography) is mandatory to support revascularization as physiologically and clinically justified and to be adjudicated as an adverse cardiac event. ACS, acute coronary syndrome; CAG, coronary angiography; CT, computed tomography; DS, degree of stenosis; FFR, fractional flow reserve; iFR, instantaneous wave-free ratio; NSTE, non-ST-segment elevation; QCA, quantitative coronary angiography; QFR, quantitative flow ratio; STE, ST-segment elevation; TLR, target lesion revascularization; TVR, target vessel revascularization

Target lesion revascularization (TLR) is defined as a repeat percutaneous intervention of the target lesion or bypass surgery of the target vessel performed for restenosis or other complication of the target lesion. Reintervention should be guided by clinically significant re-narrowing and thus includes two fundamental factors: a clinical and a functional component [i.e. fractional flow reserve (FFR), instantaneous wave-free ratio (iFR), quantitative flow ratio (QFR)]. In case of recurrent symptoms (angina pectoris) and chronic coronary syndrome, image-based non-invasive functional tests are recommended. In acute coronary syndrome, cardiac biomarkers must be assessed before revascularization. A comprehensive algorithm for the interpretation of unplanned or inter-current catheterization is provided in *Figure 1*.

The DCB ARC endorses ARC-2 support for functional assessment with invasive pressure wire (i.e. FFR, iFR), but includes angiography-derived technologies [i.e. QFR, vessel FFR, Murray law-based QFR (μQFR)] as reliable alternatives to establish the functional indication for revascularization, using the conventional cut-offs for ischaemia (i.e. FFR ≤0.80; iFR ≤0.89; QFR ≤0.80).^36^ In case of discordance between invasive physiological assessment and non-invasive testing or results on QCA, the former should take precedence in the decision-making hierarchy. When invasive functional assessment is not performed prior to revascularization, CEC adjudication, with the aid of independent QCA and QFR assessment of baseline and reintervention angiograms, is mandatory in trials in which TLR or target vessel revascularization (TVR) is an endpoint.^37^ When the epicardial physiological assessment is negative despite the presence of angina pectoris, DCB ARC suggests assessing the presence of microvascular dysfunction [i.e. index of microvascular resistance (IMR)].^38^ These measurements, in conjunction with symptoms and the results of non-invasive testing, will form the basis for event adjudication. In DCB vs. coronary artery bypass graft (CABG) trials, the ascertainment of TLR can be challenging in the CABG arm and should be based on angiography pre-bypass surgery. The ARC recommends that only TVR is considered in such trials, since surgery bypasses the target lesion.

According to the DCB ARC, planned staged procedures are not considered repeat revascularization events. However, study protocols must define the recommended time interval within which such procedures should be completed, and if this time interval is not respected (the staged procedure is performed earlier or later), the repeat revascularization should be adjudicated by the CEC. If a staged intervention is planned for a non-culprit vessel and this is performed before the scheduled time due to a readmission with symptoms, the procedure should be classified as an unplanned PCI. The ARC strongly recommends that staged procedures should not be allowed in vessels treated during the index procedure to avoid reinterventions on the index treated lesion/s.

### Thrombosis

The DCB ARC endorses the ARC-2 definition of thrombosis, with modifications to make it more specific for DCBs. Definite thrombosis needs angiographic or pathologic confirmation. Angiographic confirmation requires the presence of intracoronary thrombus that originates in the target segment (1 mm proximal or distal to the DCB applied segment) and at least one of the following criteria within a 48-h time window: (i) acute onset of ischaemic symptoms at rest; (ii) new ischaemic ECG changes that suggest acute ischaemia; and (iii) typical rise and fall in cardiac biomarkers (refer to definition of spontaneous MI). Intracoronary thrombus refers to a non-calcified spherical, ovoid, or irregular contrast-filling defect or lucency surrounded by contrast material seen in multiple views, persistence of contrast material within the lumen after washout, or visible downstream embolization of intra-luminal material.^30^ Intravascular imaging by means of optical coherence tomography (OCT, first choice) or intravascular ultrasound (IVUS) should be performed to confirm the presence of thrombus in the treated segment. Angiographic thrombosis can be defined as non-occlusive or occlusive in cases of impaired flow in the target lesion (TIMI 0-1). Small thrombi detected by OCT immediately after DCB treatment, without flow and lumen limitation, should not be considered clinically relevant, as this could be pre-existing (e.g. acute coronary syndrome), and may not lead to vessel closure and will disappear with anti-platelet therapy. Moreover, no information about their predictive value in terms of total vessel occlusion and/or MI is available.

At follow-up, thrombus detected by OCT in the appropriate clinical context (ischaemic complains, ECG alterations, and troponin release) should be considered as vessel thrombosis. Conversely, the incidental angiographic detection of an occluded target segment in the absence of the above-mentioned ancillary criteria is not considered a thrombosis, but instead is a silent target segment occlusion, which may be the chronic sequelae of a late restenotic occlusion.

Pathological confirmation requires evidence of recent thrombus within the target segment at autopsy or by the analysis of tissue retrieved following thrombectomy.

Thrombosis can be classified according to the onset time as early acute (0–24 h), early subacute (1–30 days), late (30 days–1 year), and very late (>1 year).

Probable thrombosis is defined as follows: (i) any unexplained death within the first 30 days of the DCB procedure provided the index procedure was not performed for an ST-elevation MI and (ii) irrespective of the time after the index procedure, any MI that is related to documented acute ischaemia in the territory of the DCB-treated segment, without angiographic/OCT confirmation of thrombosis, and in the absence of any other obvious cause.^30^

Similar to TLR, the adjudication of segment thrombosis is challenging due to the difficulty in identifying the DCB-treated segment at follow-up. Hence, accurate segment matching and co-localization using fiducial landmarks (i.e. side branches, bifurcations, calcifications) is warranted. For this purpose, the use of the same fluoroscopic angles and projections is recommended during the angiographic assessment of lesion thrombosis.

## Follow-up methods

The DCB ARC outlines two separate methods of follow-up:

1. Clinical and patient-level (e.g. clinical and patient-reported endpoints)
2. Procedural mechanistic (e.g. anatomical and functional)

### Clinical and patient-level follow-up

#### Composite endpoints

The adoption of composite clinical endpoints, as the combination of different individual endpoint measurements, can increase the statistical power for identifying potentially significant differences between treatments. Each individual component is conceived to reflect clinically significant events and should be reported individually. Hierarchical classification of clinical events is based on the interpretation of each individual component according to a pre-defined clinical relevance (e.g. death comes first and covers the others), while the frequency of the events describes the recurrence of each event.

Net clinical benefit composite endpoints are conceived to include different types of clinical endpoints (e.g. bleeding and ischaemic events); however, caution is necessary in their interpretation, since it may include opposite effects for safety and effectiveness.

A statistical analytical plan other than time to first event may use different hierarchical composite endpoint analyses, such as Finkelstein–Schoenfeld or win ratio. The time-to-first-event analysis only considers the first event irrespective of its severity, whereas other methods are designed to weigh both event repetition and severity, as exemplified in the GLOBAL LEADERS study.^39^

The minimum recommended clinical follow-up time when angiographic follow-up is not planned is 12 months. When angiographic follow-up is required, clinical endpoints should be collected in advance of the invasive procedure (e.g. 30 days/12 months clinical–13 months angiographic) to capture a purely clinical course not contaminated by the classical oculostenotic reflex (e.g. restenosis leading to clinically non-indicated revascularization) triggered by angiography or other objective assessment (e.g. CCTA, positron emission tomography, single-photon emission computed tomography).

In general, DCB ARC recommends clinical follow-up is extended for 5 years. When a surgical comparison is included in the study design, a longer follow-up time extended up to 10 years is recommended.

### Procedural mechanistic (anatomical and functional)

#### Invasive follow-up

##### Coronary angiography

###### Endpoints definition

Although in the setting of DES RCTs and registries late lumen loss (LLL) has been shown to best discriminate the effectiveness of treatment, with DCBs, LLL may not reflect the balance between neointimal hyperplasia (late loss), constrictive remodelling (late loss), and late expansive enlargement (late gain). Indeed, due to a vessel's elastic retraction, balloon angioplasty has a smaller acute gain compared with permanent stenting and thus a smaller LLL at follow-up. In addition, late vessel enlargement and remodelling are achievable in a non-caged vessel following DCB treatment (see Supplementary data online, *Figure S1*).^40,41^ Therefore, for DCB trials, an appropriate surrogate for parametric assessment depends on the control strategy adopted: LLL is expected to be reliable when comparing DCB vs. plain old balloon angioplasty or when comparing different types of DCBs, while net gain seems to be more informative in comparisons between DCB vs. DES (or vs. BRS). This difference appears to be more pronounced and relevant when targeting *de novo* vessels, as compared to ISR lesions.^42^ In a recent pooled analysis including ISR and *de novo* DCB trials, the use of LLL or net gain led to significant discrepancies in the interpretation of the angiographic endpoint.^42^

In-lesion QCA assessment encompasses the analysis of the treated lesion, easily recognized by means of the presence of metallic struts when a stent has been implanted. ‘In-segment QCA’ includes the treated lesion and 1 mm proximal and distal. In the DES era (ENDEAVOR III trial),^43^ ‘in-segment LLL’ showed the advantage of incorporating the edge effect (e.g. radioactive stent, actinomycin DES). Although ‘in-segment minimal lumen diameter’ is the flow-limiting anatomic parameter relevant for the patient, ‘in-segment LLL’ may not adequately reflect the neointimal inhibition of the investigated DES. ‘In-segment LLL’ incorporates the tapering effect of the vessel, artificially masking ‘in-stent LLL’, which truly reflects intrastent neointimal inhibition of the DES.

**Figure 2:**
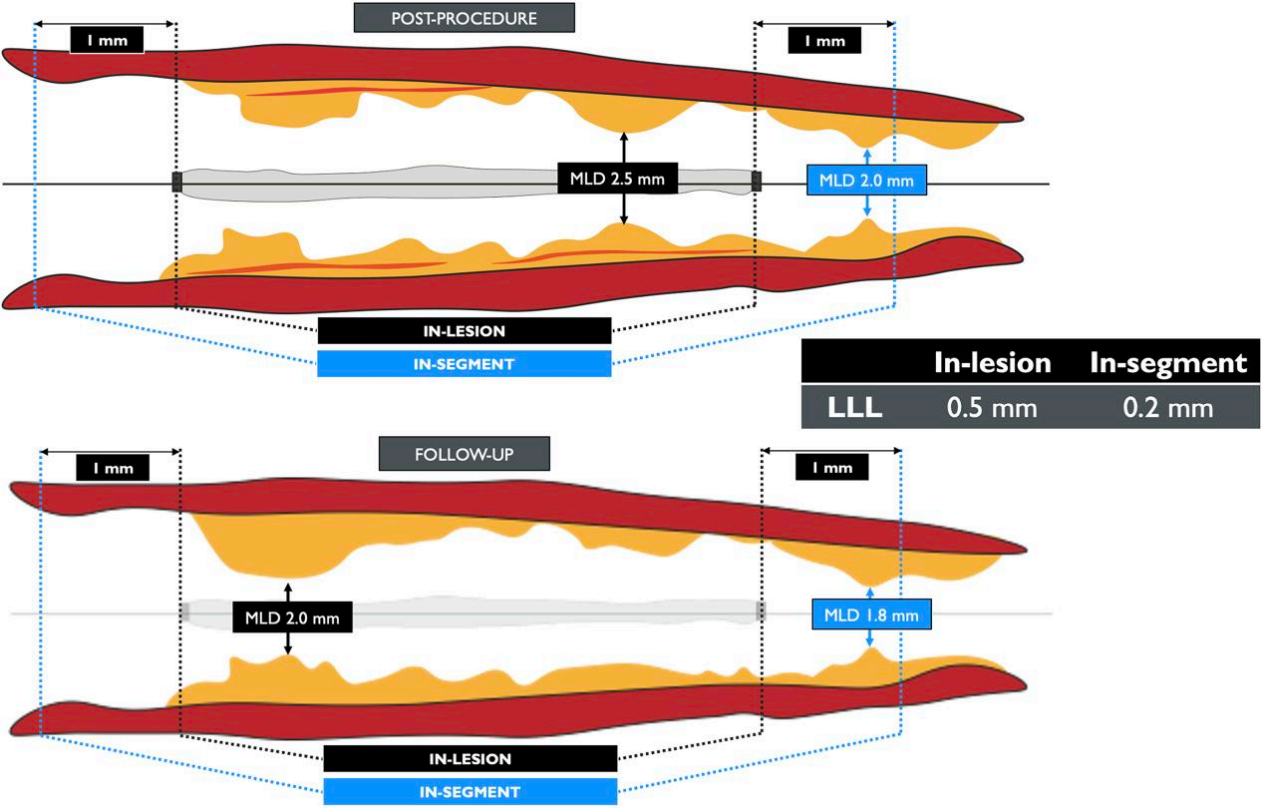
‘In-segment’ vs. ‘in-lesion’ late lumen loss in drug-coated balloon's trials. In the drug-eluting stent setting, although ‘in-segment minimal lumen diameter’ is the flow-limiting anatomic parameter relevant for the patient, ‘in-segment late lumen loss’ may not adequately reflect the neointimal inhibition of the investigated device. ‘In-segment late lumen loss’ incorporates the tapering effect of the vessel, artificially masking ‘in-stent late lumen loss’ which truly reflects intrastent neointimal inhibition of the drug-eluting stent. In the drug-coated balloon setting, ‘in-segment analysis’ provides the advantage of mitigating the risk of ‘geographical miss’ by adding a proximal and distal ‘buffer’ zone and erroneous co-localization in different time points. LLL, late lumen loss; MLD, minimal lumen diameter; mm, millimiter

**Figure 3:**
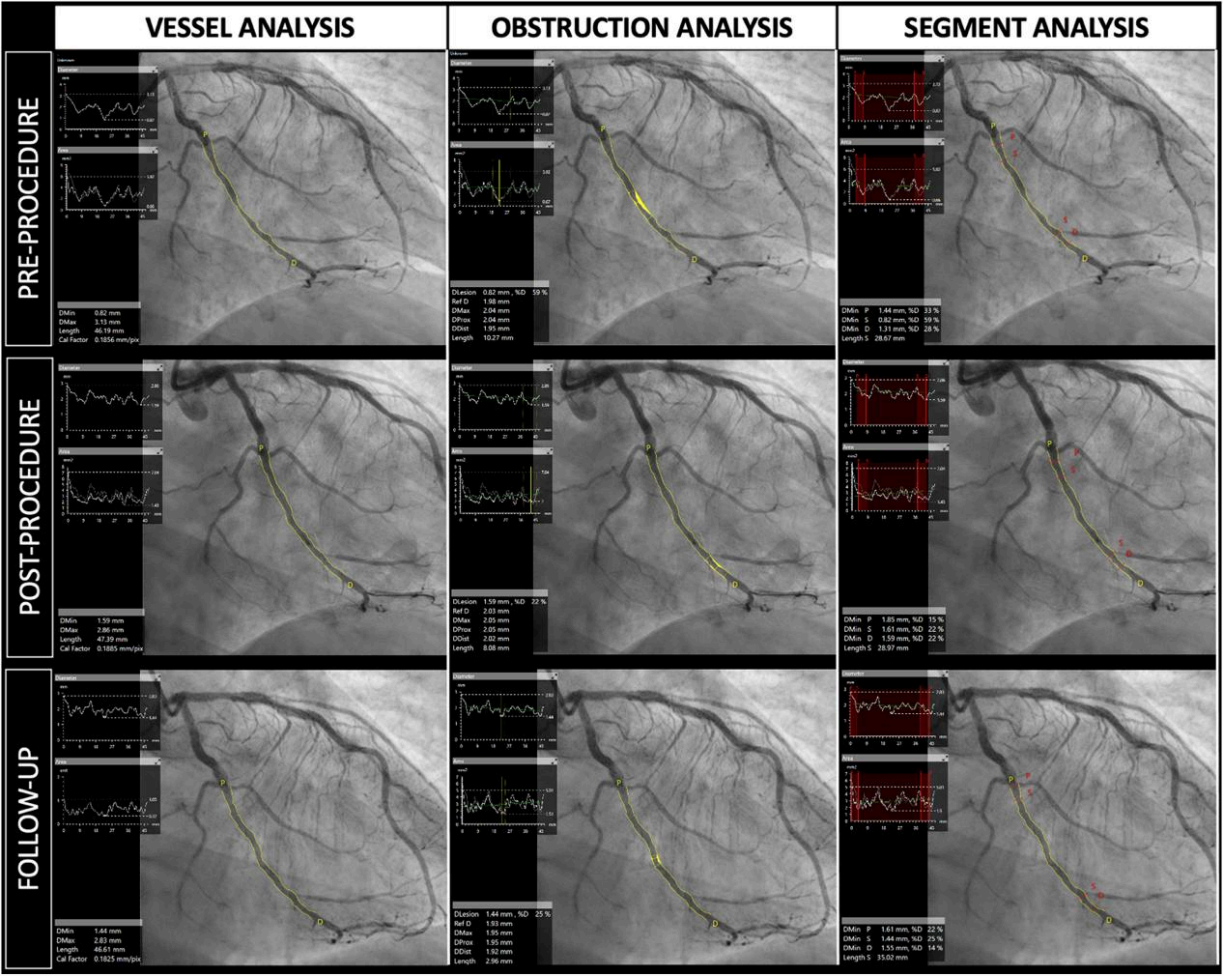
Quantitative coronary analysis in drug-coated balloon's trials. Three different quantitative coronary analysis algorithms applied to drug-coated balloon percutaneous coronary intervention: vessel analysis (left column), obstruction analysis (middle), and segment analysis (right column)

In the setting of DCB trials, ‘in-segment analysis’ provides the advantage of mitigating the risk of ‘geographic miss’ by adding a proximal and distal ‘buffer’ zone and erroneous co-localization in different time points. The DCB ARC recommends the use of ‘in-segment net gain’ in place of ‘in-segment LLL’ for clinical trials of DCB (*Figure 2*). A representative illustration of different QCA analysis protocols is provided in *Figure 3*.

###### Angiographic co-localization of the treated segment

**Figure 4:**
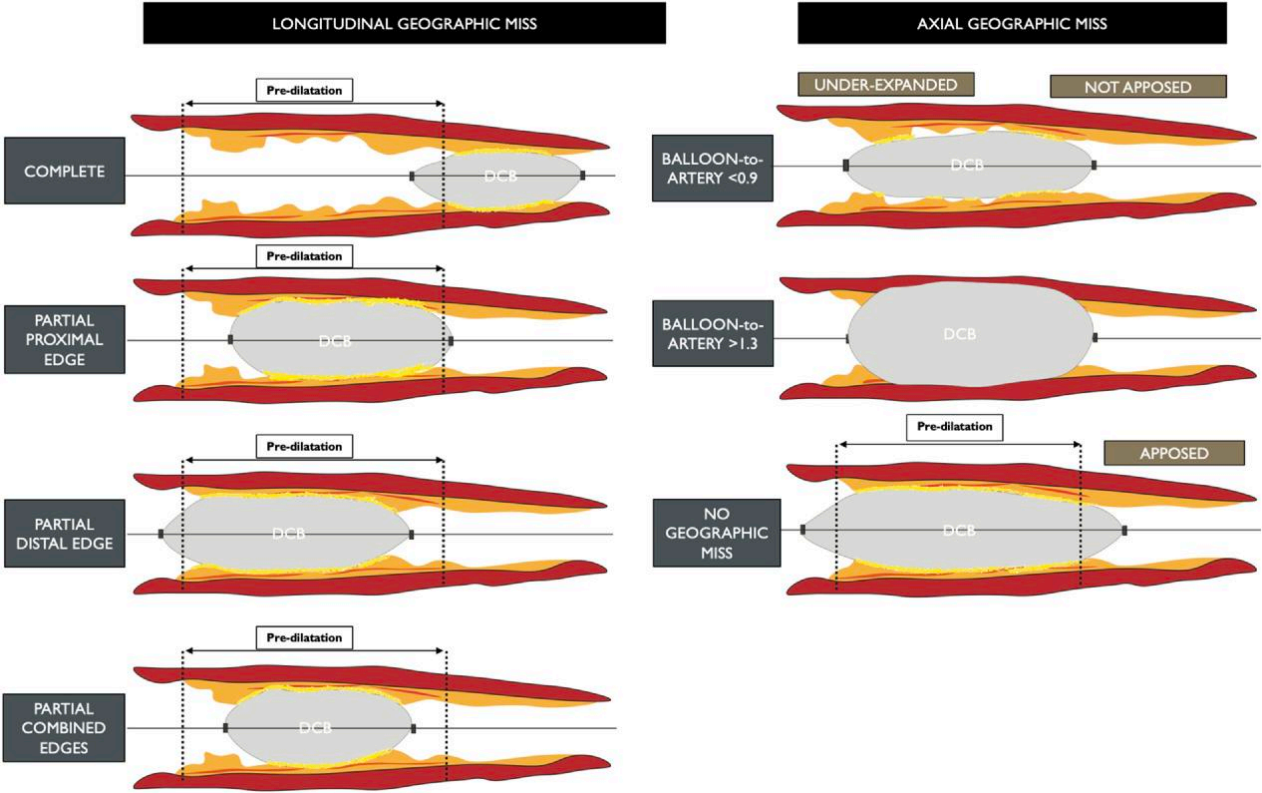
Geographic miss phenomenon with drug-coated balloons. In drug-coated balloon percutaneous coronary intervention, geographic miss can be both longitudinal (left) and axial (right). Longitudinal miss occurs due to the inflation of the drug-coated balloon not completely (complete) or only partially (partial/combined proximal and/or distal edges) covering the pre-dilated segment of the vessel. Axial geographic miss describes the use of an undersized/under expanded balloon (balloon to artery ratio < 0.9) or an oversized/overexpanded balloon (balloon to artery ratio > 1.3). DCB, drug-coated balloon

**Figure 5:**
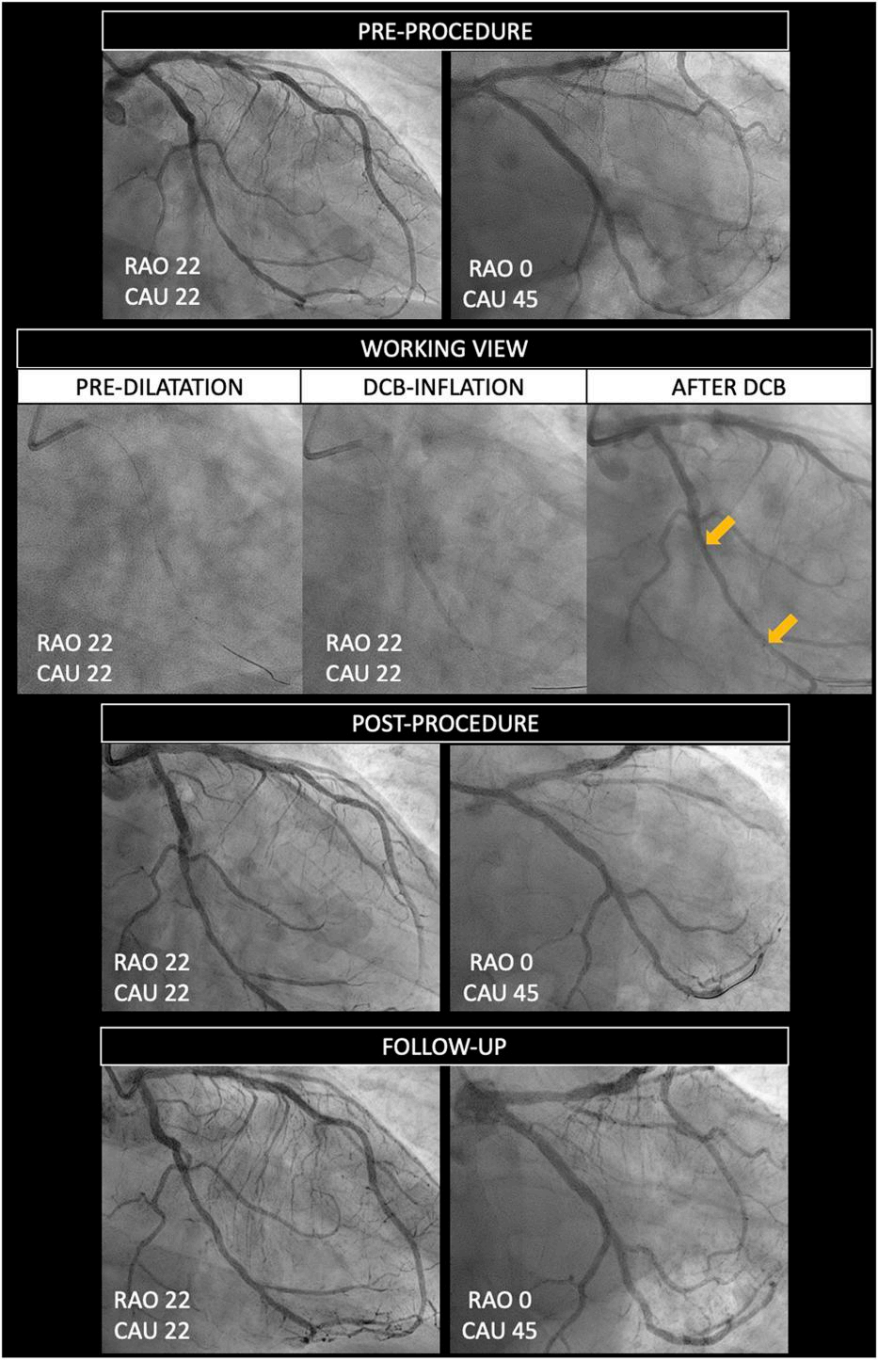
Drug-coated balloon percutaneous coronary intervention workflow in clinical trials. Two angiographic projections are acquired at baseline (pre-procedure). A working view is used during pre-dilatation and drug-coated balloon inflation. One angiographic projection (in the working angles) should be acquired immediately before and/or after deflation of the DCB, with the balloon in the same position, in order to allow precise co-localization of the device. Two final angiographic projections are required (post-procedure) using the same angles as pre-procedure. The same fluoroscopic angles and projections should also be used at follow-up. DCB, drug-coated balloon; RAO, right anterior oblique; CAU, caudal

**Figure 6:**
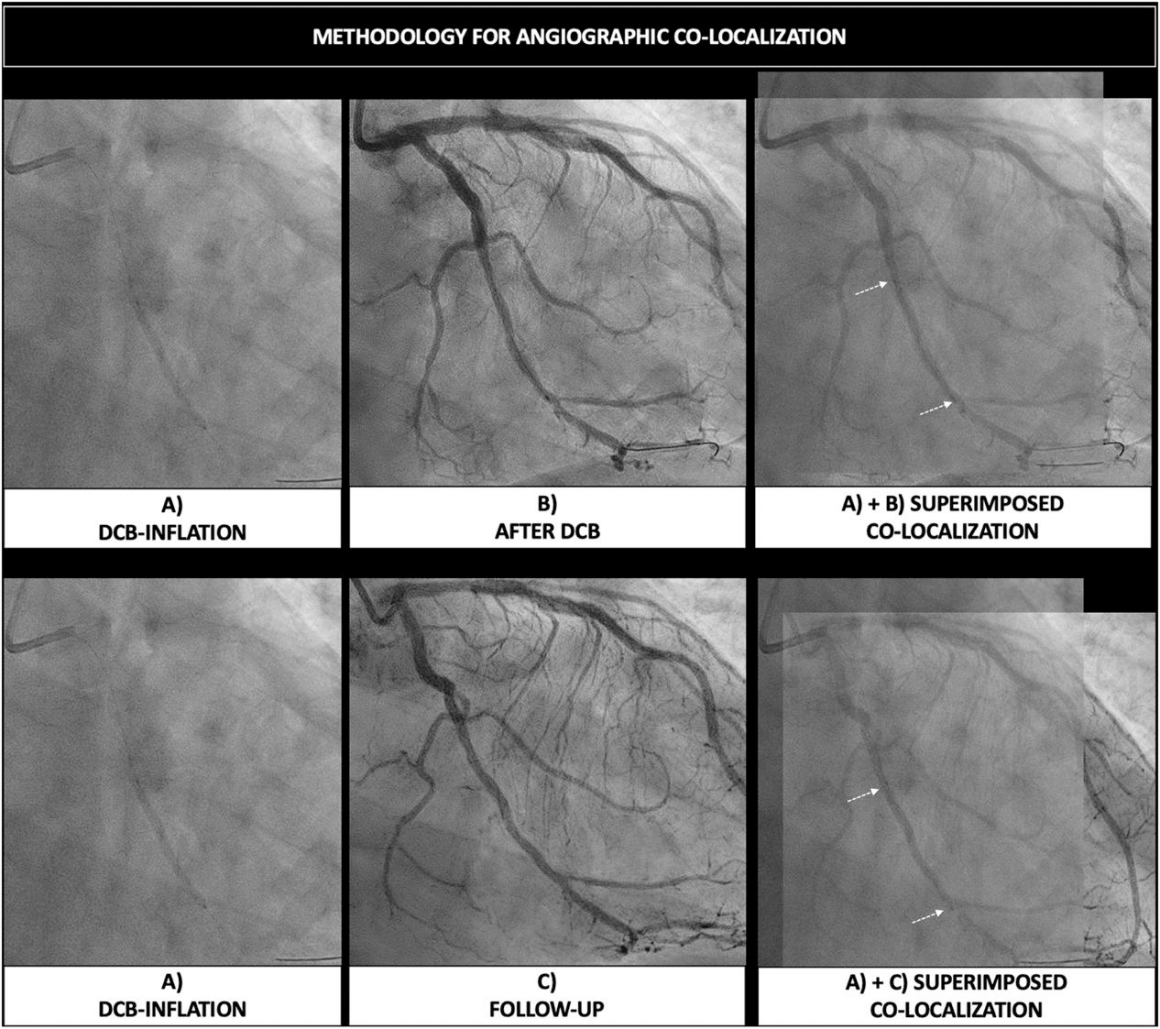
Angiographic co-localization of drug-coated balloon's treated segment in different time points. The same fluoroscopic projections allow matching and co-localization of the treated segment during drug-coated balloon inflation (*A*), after drug-coated balloon inflation (*B*), and at follow-up (*C*), by means of angiographic superimposition, which needs to be done at the same point in the cardiac cycle (preferentially end-diastole). DCB, drug-coated balloon

The absence of visible radio-opaque markers and of stent struts poses challenges in the identification of the treated segment at follow-up. Matching and co-localization of the segment that has been subject to barotrauma during pre-dilatation with the area or segment covered with the DCB is mandatory. The DCB length technically has to be longer than the length of the pre-dilatation balloon. In addition to the appropriate length, we must also ensure that the DCB is deployed within the area dilated by the pre-dilatation balloon. A comprehensive classification of geographic miss phenomenon with DCBs is provided in *Figure 4*.^44^ To assess potential geographic miss and ensure co-localization, DCB ARC recommends the acquisition of two angiographic projections at baseline (pre-procedure), with the use of one of the two throughout the procedure (during pre-dilatation and DCB inflation) as a working projection. Moreover, it recommends acquiring one angiographic projection (in the working angles) immediately before and/or after deflation of the DCB, with the balloon in the same position. Two final angiographic projections are required (post-procedure) using the same angles as pre-procedure, and these same fluoroscopic angles and projections should be used at follow-up. This process facilitates matching and co-localization of the treated segment at different time points by means of angiographic superimposition, which needs to be done at the same point in the cardiac cycle (preferentially end-diastole). A representative workflow is provided in *Figure 5*, while the concept of angiographic superimposition to match segments at different time points in order to achieve precise co-localization of the treated segment is exemplified in *Figure 6*.

**Figure 7:**
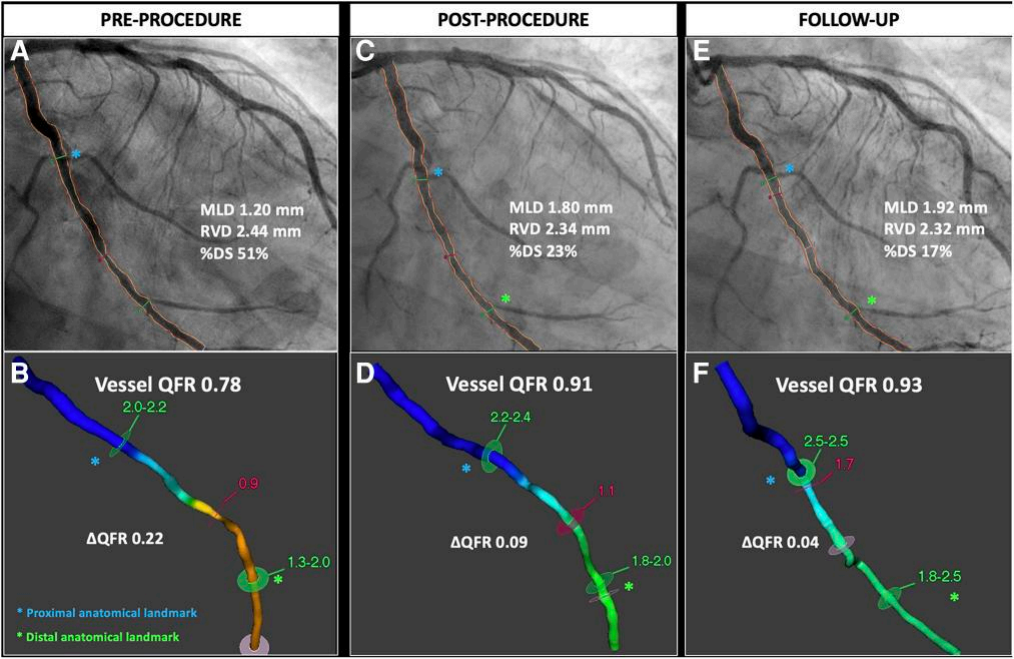
Angiography-derived quantitative flow ratio in drug-coated balloon. The use of angiography-derived fractional flow reserve technologies allows assessment of the physiological drop along the entire vessel while reaching precise co-localization of the treated segment by applying fiducial landmarks (i.e. side branches, bifurcations). Pre-procedure quantitative flow ratio depicts a flow-limiting disease (quantitative flow ratio 0.78) that improves to 0.91 after drug-coated balloon treatment. At follow-up, a non-flow-limiting quantitative flow ratio value is measured (0.93). DS, degree of stenosis; MLD, minimal lumen diameter; ΔQFR, delta QFR; QFR, quantitative flow ratio; RVD, reference vessel diameter

Moreover, the use of angiography-derived FFR technologies allows assessment of the physiological drop along the vessel (vessel QFR) and, by applying fiducial landmarks (i.e. side branches, bifurcations, calcifications), precise co-localization of the treated segment. More sophisticated techniques, such as pullback pressure gradient index (PPGi) and derivative of QFR, may be used to characterize the physiological pattern of restenosis (focal vs. diffuse)^45,46^ (*Figure 7*). In device comparison studies, the assessment of microvascular resistance and function could be beneficial, allowing detection of sub-clinical microvascular damage potentially related to the procedure. In this respect, novel angiography-derived technologies and computations could provide a valid, reproducible, widely available, and fast computational assessment, with no need for dedicated guidewires or hyperaemia.^47,48^

###### Optical coherence tomography/intravascular ultrasound co-localization of the treated segment

Automatic co-registration of OCT/IVUS and coronary angiography allows precise co-localization of the anatomical and imaging data,^49^ and can be performed online (Syncvision, Philips Corporation; OPTIS Integrated System, Abbott) or retrospectively offline (AngioPlus Core and OctPlus, Pulse Medical Imaging Technology).^50^ This is of potential relevance in the DCB field by matching the segment that underwent lesion preparation and subsequent DCB treatment with the same segment visualized at follow-up to detect any possible geographic miss. The role of co-registration in assessing lesion and dissection healing, and vessel remodelling is still under investigation. Intravascular ultrasound, which does not require contrast injection for visualization, theoretically appears to be safer than OCT, which necessitates clearing the vessel from blood through contrast injections. Currently, there is no evidence indicating an increase in dissections with the use of OCT.

Non-invasive follow-up and CCTA are described in the Supplementary data.^51–59^

## Statistical consideration

Statistical considerations related to the analytical plan (intention-to-treat, per-protocol, and as-treated analyses) and to composite endpoint and repeated events interpretation (Finkelstein, win ratio analysis) are reported in the Supplementary data and *Figure S2*.^60–65^

## Lesions and clinical settings for drug-coated balloon treatment

An overview of the evidence and indications of the use of DCBs in different lesions and clinical settings are summarized in the Supplementary data, *Tables S1–S6* and *Figure S3*.^1,4,15,18,19,21,35,45,56,66–92^ Relevant definitions are summarized in *Table 6*.

**Table 6:** Definitions of lesions and clinical settings for drug-coated balloon treatment

	Nomenclature	Description
1	In-stent restenosis (ISR)	A diameter stenosis >50% in the stented segment or within a 5 mm proximal or distal margin Avoid the inclusion of patients with acute MI (<72 h) and very early (<1 month) ISR
2	Small vessels Very small vessels	Reference vessel diameter (RVD) < 2.75 mm Lesion length <25 mm Reference vessel diameter (RVD) < 2.25 mm
3	Late lumen enlargement Positive vessel remodelling	Negative lumen loss CCTA: outer vessel diameter > 10% of the reference normal segment in the same vessel (remodelling index >1.1) IVUS: >5% difference in the external elastic membrane cross-sectional area at the site of plaque compared to a non-diseased reference segment
4	Diffuse disease	Coronary segment ≥25 mm in length, with vessel wall irregularities and no clear focal lesion
5	Large vessels	Reference vessel diameter (RVD) ≥ 2.75 mm Lesion length <25 mm
6	Calcified lesions	Angiographic appearance of radiopacities without cardiac motion before contrast injection affecting both sides of the arterial wall (tramway-track appearance)
7	Chronic total occlusion	Occlusion with the absence of antegrade flow with a documented (definite CTO) or presumed (probable CTO) duration of ≥3 months
8	Bifurcations	Coronary artery narrowing occurring adjacent to, and/or involving, the origin of a significant side branch (SB), anatomically represented by complex vessel/function structure composed of three different vessel segments (proximal main vessel, distal main vessel and SB)
9	High bleeding risk	1-year risk of BARC 3 or 5 bleeding ≥4% or of an intracranial haemorrhage ≥1% ARC-HBR proposed 20 clinical criteria and patients are at HBR if at least one major or two minor criteria are met

ARC, academic research consortium; BARC, bleeding academic research consortium; CCTA, coronary computer tomography angiography; CTO, chronic total occlusion; HBR, high bleeding risk; ISR, in-stent restenosis; IVUS, intravascular ultrasound; MI, myocardial infarction; RVD, reference vessel diameter; SB, side branch.

## Supplementary Material

ehaf029_Supplementary_Data

## Declarations

### Data Availability

No data were generated or analysed for this manuscript.
