# Supplementary material for: Definitions and standardized endpoints for the use of drug-coated balloon in coronary artery disease: consensus document of the Drug Coated Balloon Academic Research Consortium
Source: Eur Heart J. 2025 Apr 24;46(26):2498–519. doi: 10.1093/eurheartj/ehaf029 (PMC12232911; doi:10.1093/eurheartj/ehaf029)
Supplement: ehaf029_Supplementary_Data [file ehaf029_supplementary_data.docx]

**Definitions and standardized endpoints for the use of drug coated balloon in coronary artery disease: consensus document of the Drug Coated Balloon Academic Research Consortium**

***<<Supplementary material>>***

Simone Fezzi MD Msc^1,2^, Bruno Scheller MD^3^, Bernardo Cortese MD PhD^4,5^,

Fernando Alfonso MD PhD^6^, Raban Jeger MD^7^, Antonio Colombo MD^8^, Michael Joner MD PhD^9^, Eun-Seok-Shin MD PhD^10^, Franz Kleber MD PhD^11^, Azeem Latib MD^12^,

Tuomas T. Rissanen MD PhD^13^, Simon Eccleshall MD^14^, Flavio Ribichini MD^2^,

Ling Tao MD PhD^15^, Bon-Kwon Koo MD PhD^16^, Alaide Chieffo MD PhD^17,18^, Junbo Ge MD^19^, Juan F. Granada MD^20^, Hans-Peter Stoll MD PhD^21^, Christian Spaulding MD PhD^22^,

Rafael Cavalcante MD^23^, Alexandre Abizaid MD PhD^24^, Takashi Muramatsu MD PhD^25^, Konstantinos Dean Boudoulas MD^26^, Ron Waksman MD^27^, Roxana Mehran MD^28^,

Donald Cutlip MD^29^, Mitchell Krucoff MD^30^, Gregg W. Stone MD^28^, Scot Garg MD PhD^31,32^, Yoshinobu Onuma MD PhD^1^, Patrick W. Serruys MD PhD^1^*

^1^ Department of Cardiology, University of Galway, Galway, Ireland.

^2^ Division of Cardiology, Department of Medicine, Verona University Hospital, Verona, Italy.

^3^ Clinical and Experimental Interventional Cardiology, University of Saarland, Homburg/Saar, Germany.

^4^ Fondazione Ricerca e Innovazione Cardiovascolare, Milan, Italy

^5^ DCB Academy, Milan, Italy ^6^ Department of Cardiology, Hospital Universitario de La Princesa, Universidad Autónoma de Madrid. IIS-IP, CIBERCV, Madrid, Spain.

^7^ Triemli Hospital Zürich, Switzerland, and University of Basel, Basel, Switzerland.

^8^ Cardio Center, Humanitas Clinical and Research Hospital IRCCS, Rozzano, Milan, Italy.

^9^ Department of Cardiology, German Heart Center Munich, Technical University of Munich, Munich, Germany.

^10^ Department of Cardiology, Ulsan University Hospital, University of Ulsan College of Medicine, Ulsan, South Korea

^11^ Mitteldeutsches Herzzentrum, University Halle-Wittenberg, Halle, Germany.

^12^ Department of Cardiology, Montefiore Medical Center, Bronx, New York, USA.

^13^ Heart Center, Central Hospital of North Karelia, Siunsote, Joensuu, Finland.

^14^ Department of Cardiology, Norfolk and Norwich University Hospital, Norwich, United Kingdom.

^15^ Department of Cardiology, Xijing Hospital, Fourth Military Medical University, No. 15 Changle West Road, Xi’an, China.

^16^ Department of Internal Medicine and Cardiovascular Center, Seoul National University Hospital, Seoul National University of College of Medicine, Seoul, South Korea.

^17^ Department of Medicine, Vita Salute San Raffaele University, Milan, Italy.

^18^ Interventional Cardiology, IRCCS San Raffaele Scientific Institute, Milan, Italy.

^19^ Zhongshan Hospital, Fudan University, Shanghai, China.

^20^ Cardiovascular Research Foundation, Columbia University Medical Center, New York, USA.

^21^ Clinical Research, Biosensors Clinical Research, Morges, Switzerland.

^22^Department of Cardiology, European Hospital Georges Pompidou, Assistance Publique Hôpitaux de Paris and INSERM, Paris, France.

^23^ Boston Scientific, Marlborough, Massachusetts, USA.

^24^ Instituto do Coracao, Hospital das Clinicas, Faculdade de Medicina, Universidade de Sao Paulo, Sao Paulo, Brasil.

^25^ Department of Cardiology, Fujita Health University Hospital, Toyoake, Japan.

^26^ Division of Cardiovascular Medicine, The Ohio State University, Columbus, Ohio, USA.

^27^ Section of Interventional Cardiology, MedStar Washington Hospital Center, Washington, District of Columbia, USA.

^28^ The Zena and Michael A. Wiener Cardiovascular Institute, Icahn School of Medicine at Mount Sinai, New York, USA.

^29^ Beth Israel Deaconess Medical Center and Harvard Medical School, Boston MA, USA.

^30^ Department of Medicine, Duke University School of Medicine, Durham, USA.

^31^ Department of Cardiology, Royal Blackburn Hospital, Blackburn, United Kingdom.

^32^ School of Medicine, University of Central Lancashire, Preston, United Kingdom.

***Address for correspondence:**

Patrick W. Serruys, MD PhD

Established Professor of Interventional Medicine and Innovation, University of Galway, Galway, Ireland.

University Road, Galway, H91 TK33, Ireland.

Tel: +353 91 524411

E-mail: [patrick.w.j.c.serruys@gmail.com](mailto:patrick.w.j.c.serruys@gmail.com)

1. **Types of clinical studies in DCB**
2. ***First-in-human studies***

Studies in the DCB field investigating for the first time a new balloon, drug, or coating to improve transfer capabilities, vessel wall penetration or extend local drug retention in human applications are referred to as a “First-in-Human” (FIH) or “First-in-Man” (FIM), and are aimed at providing preliminary information on device feasibility (proof of principle) in humans whilst identifying any major safety concerns. Consequently, their design should primarily be single-arm with outcomes reported through descriptive statistics, and as their intent is to expose only a minimal number of subjects to the new device as they are typically not powered for specific endpoints and include sample sizes of <100 patients. They are expected to provide clear pre-specified “stopping” rules to prevent the exposure of further patients to futile or dangerous treatments. These studies are appropriate early in device development when initial clinical experience is necessary because non-clinical testing methods are neither available nor adequate to provide the information needed to advance product development. As with all clinical studies, initiation of an early feasibility study must be justified by an appropriate benefit-risk analysis and adequate human subject protection measures^1^. Following FIH studies, the efficacy of the technology should be investigated in clinical registries or small randomized clinical trials, usually designed to have mechanistic endpoints and to compare performance with predefined “Objective Performance Criteria” (OPC)^1^.

In the case of DCBs, several technologies have been tested for in-stent restenosis (ISR) or de-novo lesions in coronary arteries. For the latter, the limited available evidence has mainly been accrued in small-vessel disease. Future dedicated studies should target the application of different DCB technologies in distinct lesion types (i.e., de-novo, large vessels, bifurcations, complex lesions including chronic total occlusions and left-main) and/or specific clinical patient conditions (i.e., high bleeding risk, diabetes mellitus, acute coronary syndromes).

1. ***Studies for regulatory approval***

FIH studies, conducted as the first step in generating evidence, are not sufficient for regulatory approval, and must be supplemented by larger pivotal studies preferably powered for clinical endpoints of safety and performance (efficacy). Surrogate endpoints can be embedded as ancillary and/or in support of clinical endpoints.

Currently, in the European Union, to obtain a CE-mark (CE = Conformité Européene) for a new device under the Medical Device Regulation (MDR), an EU-pivotal study is needed that typically characterizes the new device through mechanistic safety and efficacy endpoints, or composites. These studies can be randomized or single arm in design and seek to compare the outcomes of the experimental arm with the contemporary standard of care (control group) through superiority or non-inferiority comparisons. For single-arm designs, comparison with historical trial data or an OPC can be used, once medically and statistically justified^1^. CE-mark certificates for a short period (e.g., 2 years) can be granted once safety is confirmed, in the presence of limited additional clinical evidence from surrogate endpoints with short follow-up (e.g., 6 months), limited samples size and/or non-randomized designs. Granting certificates with longer durations (e.g., 5 years) requires pivotal studies with clinical primary endpoints, evaluated at appropriately long follow-up (12 months), with adequate sample sizes delivering more substantial evidence. The ultimate goal of these more comprehensive clinical outcome trials is to more reliably detect potential safety signals not identified in the initial surrogate endpoint study. In non-inferiority trials, sample sizes are driven by the need for a smaller non-inferiority margin, that still should be clinically acceptable.

Alternatively, in the United States, the manufacturer needs an investigational device exemption (IDE) to conduct significant risk, pre-approval device trials^2^, which are typically clinical-outcomes trials, with large sample sizes. Another major difference to other regions is that in the United States the trial design may be discussed and developed jointly with the Food and Drug Administration (FDA), which may subsequently facilitate final approval once the trial has been completed and published.

The specific principles and requirements for approval by the Chinese Food and Drug Administration (CFDA) and the Japanese Pharmaceuticals and Medical Devices Agency (PMDA) are beyond the scope of this document^3,4^. The DCB ARC endorses the “Harmonization by Doing" (HBD) approach for medical device regulation, which is employed by the FDA and the PMDA. This aims to achieve regulatory harmonization and streamline the evaluation and approval processes for medical devices, supporting a practical collaboration and ongoing communication between regulatory authorities, industry stakeholders, and other relevant parties, to reduce duplicative efforts, minimize regulatory barriers, and enhance the efficiency of the regulatory review and approval processes.

The DCB ARC recommends that trials comparing the performance of DCBs to plain old balloon angioplasty (POBA) should be designed to show superiority of the DCB, whereas when comparing DCBs to DES or alternatively approved DCB, the study design should aim to show, at the very least, non-inferiority of the DCB.

1. ***Sham procedure studies***

Clinical endpoints with a subjective component, quality-of-life assessments, or patient-reported outcomes, have recently gained increasing attention and importance in the cardiovascular field. However, phenomena like the Hawthorne Effect (patients behave differently once they feel observed) or the Pygmalion Effect (high expectations led to better outcomes and vice versa) have the potential to hamper such assessments, and therefore, if feasible, trial designs utilising placebo assignments and “blinding” would be preferrable^5^. “Sham procedures” can be used as the placebo arm of randomised control trials (RCT) of medical devices^6^, whilst a double-blind trial design is critical in trials aiming to test subjective outcome metrics.

Although DCB ARC acknowledges the relevance of sham-controlled trials in the setting of CCS, by comparison of DCB treatment with optimal medical therapy (OMT)^7^, it does not recognize a clear need for sham procedure studies in the DCB field. Performance of sham-controlled trials is challenging and may be also controversial from an ethical stand point. Careful study design, in terms of sample size, primary endpoint and duration of follow-up is paramount. ﻿

1. ***Clinical endpoints***

**Myocardial infarction**

*Postprocedural cardiac biomarkers rise*

DCB ARC suggests reporting any rise in cardiac biomarkers occurring after a minimum of 6 hours following the end of the procedure, even if they do not meet the criteria for a PMI. In trials comparing devices, the detection of differences in the rise of cardiac biomarkers, even if not clinically significant, may be of relevance in the adjudication of their safety profile^8^.

Post-procedural cardiac biomarkers rise should be classified according to the angiographic findings as related to angiographic complications (type 1) or not (type 2). Angiographic complications should be recorded as transient (intraprocedural) or persisting at the end of the procedure, as shown in **Table 3**. For this purpose, the accurate report of intraprocedural complications, including transient ones, is relevant and has been linked to adverse short-term outcomes in patients undergoing PCI^9^.

*Spontaneous myocardial infarction*

Spontaneous MI is defined according to the 4^th^ universal definition of MI (UDMI; Type 1, 2, 3, 4b or 4c)^10^. ARC-2 already reported the difficulty in differentiating between Type 1 and Type 2 MI or non-ischemic myocardial injury from necrosis, as additional investigations that might allow clarification, are usually judged inappropriate from a clinical perspective.

Prior or silent/unrecognized MI ﻿is defined as abnormal Q waves with or without symptoms in the absence of non-ischaemic causes, imaging evidence of loss of viable myocardium in a pattern consistent with ischaemic etiology, or pathoanatomical findings of a prior MI. DCB ARC suggests that “prior or silent/ unrecognized MI” should not be included in the primary endpoint adjudication, as no proof of cardiac biomarker elevation is available.

Target-lesion MI is defined as any MI associated with angiographic confirmation that the culprit lesion corresponds to the DCB treated segment (1 mm proximal and distal to the balloon). Any MI attributed to the target vessel, but not involving the target lesion should be defined as “target-vessel non-target lesion MI”.

**3. Follow-up methods**

1. **Clinical and patient-level follow-up**

**Patient-, site-, central adjudication–reported and cost-effectiveness endpoints**

The role of coronary revascularization in preventing hard adverse cardiac events (MI, death), especially in the setting of CCS, has been recently questioned^11^. However, in CCS revascularization plays an important role in reducing symptoms, and improving functional status, and quality of life (QoL). Traditional clinical outcome measures (laboratory tests, mechanistic outcomes), may not fully capture from a patient's perspective their experience of the treatment^12,13^. PROMs are becoming increasingly relevant as they provide valuable insights into a patient's QoL, symptoms, and treatment satisfaction. Several methods to assess PROMs are available, such as questionnaires [Seattle Angina Questionary (SAQ) 19 and 5, EuroQol 5D, SF-36, SF-12], interviews, and electronic health records. PROMs are especially valuable in chronic conditions, where the impact of treatment on a patient's daily life is significant. For this purpose, the measurement of quality-adjusted life-years (QALY) in cases of long, or very-long term follow-up, is preferred combining hard clinical events and QoL improvement. The QALY metric is also used in cost-effectiveness analyses to compare the costs and benefits of different interventions, helping to prioritize healthcare resources (e.g., EXCEL trial)^14^.

Adjudication of PROMs, however, can pose several challenges, as patients may not have the ability or willingness to accurately self-report their experiences. Additionally, the validity and reliability of PROMs may be impacted by bias (recall bias, social desirability bias, self-report bias). To overcome these challenges the assessment of PROMs must be designed and executed to ensure the accuracy and validity of the collected data, providing ﻿psychometric properties (validity, reliability, responsiveness, and interpretability) proven to measure the intended domain^12^. Therefore, the utilization of digital health technologies, such as the incorporation of cartoon or graphic representations, holds the potential to enhance the willingness and ability of patients to provide accurate self-reported PROMs. Double blinded assessment with or without the ancillary use of a sham procedure, is particularly valuable in ensuring PROMs accuracy and validity.

EuroQol-5D (EQ-5D) and SF-36 are commonly used PROMs in clinical trials. The EQ-5D is a standardized instrument used to measure health-related QoL based on five dimensions (mobility, self-care, usual activities, pain/discomfort, and anxiety/depression), while the SF-36, with a shortened version (SF-12), is a broader tool that measures both physical and mental health-related QoL^15^.

﻿PROMs play a key role in studies investigating the clinical benefit derived from different pharmacological regimens and sham-controlled studies, when the two competing comparators might lead to significant differences in patient’s perceived health status, and not in those comparing strategies or devices.

1. **Procedural mechanistic (anatomical and functional)**

**Noninvasive follow-up**

***CCTA***

CCTA provides high diagnostic accuracy for detecting obstructive stenoses in patients with suspected CAD^16^. It enables a comprehensive assessment of the epicardial conductance vessels by analyzing the lumen, plaque composition and the functional characteristics of lesions and vessels. In the follow-up of vessels that underwent PCI, it provides a net advantage by avoiding an invasive procedure and the related risks to the patient. Nevertheless, metallic stents hamper its diagnostic accuracy due to the occurrence of a blooming artifact generated by the metal^17^. Conversely, CCTA was seen to yield good diagnostic accuracy compared to IVUS during the follow-up ﻿of patients treated with bioresorbable technologies^18,19^. Notably, CCTA seems to be of potential use during follow-up after DCB treatment, overcoming potential drawbacks related to the invasive assessment of severely calcified or tortuous vessels and allowing the definition of the amount of plaque, vessel remodeling and functional evaluation. On top of anatomic surrogate endpoints, such as minimum lumen area (MLA), plaque burden (PB) and vessel remodeling, CCTA potentially allows assessment of plaque composition and vulnerability (i.e., low-attenuation plaque, positive remodeling, spotty calcifications, napkin-ring sign). Moreover, the distribution of physiological values along the studied vessel can be analyzed. In particular a distal FFR-CT value can be measured, as well as the physiological drop across the treated segment (ΔFFR-CT). The adoption of these methodologies is growing and could be of potential application in future DCB trials, pending greater availability in different participating centers.

However, CCTA analysis is particularly susceptible to “geographical miss”, with co-localization of the treated segment challenging (with coronary angiography). The use of fiducial co-localization (e.g., side branches) can overcome such limitations, especially when functional assessment along the vessel is performed. CCTA analysis may also be limited in very small vessels (<2.0 mm)^16^.

1. **Statistical consideration**

**Analytical plan (intention-to-treat, per-protocol, and as-treated analyses)**

DCB ARC recommends specifying up-front the analytical plan to be used in DCB trials, which is largely dependent on the study design. As a class effect is not anticipated for DCBs, an appropriate sample size and non-inferiority margin, is recommended.

In this regard, the interpretation of cross-over and adjudication is crucial. According to the intention-to-treat (ITT) principle, statistical analyses are conducted according to the group to which patients are randomized, regardless of whether they actually received the intervention or adhered to the protocol. ITT is always accompanied by a per-protocol analysis, which aims to ensure the intrinsic comparability of the two arms (comparators) to detect true differences. In the specific setting of DCB trials, especially in “device-comparing” or in the “leave nothing behind” strategy-comparing studies, an ITT analysis may result in inaccurate conclusions, and a different analytical plan may better reflect the comparison between the two arms (i.e., Per-protocol, As-treated). Hence, ITT is reliable whenever a “cross-over” or “blended” strategy therapy is allowed.

According to the per-protocol analysis plan only those participants who received the assigned intervention (per randomization) and strictly followed the specific protocol for the device are included in the statistical analysis, better estimating the true treatment effect. According to the as treated plan, participants are analyzed based on the treatment they actually received, rather than the treatment to which they were allocated by randomization^20^.

Novel trials designs should be also considered. The adaptative trial design, often incorporating Bayesian analyses of the data, represents a dynamic approach that allows for flexibility and responsiveness during a study. Pre-defined interim analyses with pre-defined statistical penalties, enable informed decisions to be made about the trial's course as data accumulates.

Bayesian analysis is particularly useful when dealing with small sample sizes, complex models, and situations where prior knowledge or expert opinions are relevant, by providing a coherent framework for incorporating both existing knowledge and new data to make probabilistic inferences.

Event-driven clinical trials, exemplified by the PROSPECT trial design^21^, introduce an additional layer of adaptability. In these trials, enrollment continues until a predefined event rate or outcome is reached. Once this threshold is achieved, the trial is halted, ensuring that the study's conclusions are drawn from a sufficient number of events, potentially providing valuable clinical data.

**Statistical approach related to composite endpoint and repeated events interpretation (Finkelstein, Win-ratio analysis)**

Composite endpoint interpretation is influenced by the statistical approach that is used, and the choice relies on the study design, the nature of the composite endpoint, and the objectives of the analysis. Time-to-first-event analysis treats all the components of the composite endpoint as having equal relevance and considers the first event occurring during follow-up as the most important. This statistical approach is considered the standard method for the analysis of composite endpoints, being simple to perform and easy to understand, providing a straightforward interpretation of the result. ﻿However, non-fatal events that occur early have more impact than more serious events (i.e., stroke or death) occurring later. Alternative methods, such as win ratio analysis, Cox-based models for recurrent events, or weighted cumulative events (WCE) analysis, are designed to weigh the number (repeated events) and the severity of each event, which may provide a more complete and accurate assessment of the treatment effect. The win ratio analysis and Cox-based models for recurrent events consider all the events occurring during follow-up and incorporates the severity of the clinical events by assigning different weights to different types of events. The WCE analysis is a more complex method that considers the total burden of events and assigns different weights to different types of events based on their clinical significance. This method considers the timing, type, and frequency of all events occurring during follow-up and provides a more comprehensive assessment of the treatment effect^22^.

The application of these statistical plans may empower clinical trial interpretation, especially when individual components of the composite endpoints seem to vary substantially in severity and timing. Such methods should be used as pre-specified secondary analyses, according to patient type, and the devices and strategies used. The ranking and severity of each event should be pre-defined in the study design^23^. A decision tree for statistical models is illustrated in **Supplementary Figure 2**.

﻿The sample size calculation should be based on the primary analysis, having time-to-first-event analysis as reference. When recurrent events and/or event severity are used, simulation techniques and dedicated codes are required for sample size calculations^24,25^.

1. **Lesions and clinical settings for DCB treatment**
2. ***In-stent restenosis***

The main evidence-based indication for using a DCB is to treat ISR, and according to many RCTs and meta-analyses DCBs are superior to conventional POBA, BMS, and first-generation DES, and comparable to new generation DES in the management of ISR^26^. Although acute and late angiographic findings tend to be superior with DES over DCB, clinical safety and efficacy are largely comparable^27,28^. A comprehensive description of the study design, angiographic and clinical endpoints of clinical trials evaluating the performance of DCB in the setting of ISR is provided in **Supplementary Table 1**.

ISR is defined as ﻿a diameter stenosis (DS%) >50% in the stented segment or within a 5 mm proximal or distal margin^29^, and when associated with angina and/or documented ischaemia, repeat revascularization is indicated.

﻿The American College of Cardiology/American Heart Association lesion classification and the widely used Mehran’s angiographic ISR classification provide useful tools to determine acute procedural results and the long-term angiographic outcome of patients with ISR (**Supplementary Table 2**)^30-32^.

Intracoronary imaging plays a pivotal role in tackling ISR by helping determine the mechanistic causes of the restenosis other than intimal hyperplasia, such as chronic under-expansion (18–40%), stent fracture (<5%), and neoatherosclerosis.

Intravascular ultrasound (IVUS) ISR classification and optical coherence tomography (OCT) based ISR mechanisms classification are presented in **Supplementary Table 2**.

DCB ARC recommends determining the angiographic and intracoronary imaging-defined pattern of ISR, as these are the major predictors of recurrent restenosis and subsequent reintervention^33^.

To avoid the inclusion of patients presenting with stent thrombosis, DCB ARC recommends excluding patients with acute MI (<72H) and very early (<1 month) ISR.

1. ***De novo lesions***

﻿The rationale of using DCBs for the treatment of *de novo* CAD is to prevent restenosis by releasing anti-proliferative drug into the arterial wall, without permanent vessel caging. Although this strategy has yielded non-inferior results in selected lesions compared to DES in several RCTs, it is yet to be endorsed by international guidelines^34,35^.

- ***Small vessels***

﻿Coronary arteries with a reference vessel diameter (RVD) <2.75 mm, as assessed by coronary angiography are defined as “small vessels” and this criterion is used by most RCTs of DCBs in small vessels; although notably the BASKET-SMALL 2 study used a <3 mm cut-off criteria^35,36^. Very small vessels are usually defined as those with an RVD <2.25 mm^37^, whilst universally, the minimum RVD required for treatment is ≥2 mm. In order to differentiate between trials of small vessel and diffuse disease, DCB ARC suggests a lesion length <25 mm as the cut-off for eligibility.

﻿Assessment of vessel size should be performed after administering intracoronary nitroglycerin to maximize RVD, regardless of which imaging modality is used. Intravascular OCT enables the most precise assessment of target vessel size, with angiography associated with several degrees of under-estimation, and IVUS overestimation^38^. PCI in small vessels disease is hampered by an ﻿inverse relationship between vessel diameter and the future risk of restenosis^39^.

A comprehensive description of study design, angiographic and clinical endpoints of clinical trials evaluating the performance of DCB in the setting of de novo small vessels is provided in **Supplementary Table 3.**

**Late lumen enlargement**

DCB ARC defines angiographic late lumen enlargement as negative late lumen loss. Positive remodeling is defined on CCTA as an outer vessel diameter > 10% of the reference normal segment in the same vessel (remodeling index >1.1), or on IVUS by a >5% difference in the external elastic membrane cross-sectional area at the site of plaque compared to a non-diseased reference segment^16^.

- ***Diffuse disease***

﻿Diffuse disease is defined as a coronary segment ≥25 mm in length, with vessel wall irregularities and no clear focal lesion^40^. DCB ARC suggests a lesion length ≥25 mm, either determined by quantitative coronary angiography or by intravascular imaging, as the cut-off for eligibility in clinical trials of diffuse disease. Adequate views should be use to prevent angiographic foreshortening of lesion length.

- ***Large vessels and left main***

Large coronary vessels are defined by an RVD ≥2.75 mm, as assessed by coronary angiography. DCB ARC suggests a lesion length <25 mm as cut-off to differentiate trials of large vessels from diffuse disease (≥25mm). To date, there has been no dedicated RCTs of DCBs in large vessels.

Clinical trials assessing the performance of DCB in the setting of de novo large vessels are summarized in **Supplementary** **Table 4.**

- ***Calcified lesions (calcium debulking devices)***

***﻿***Severe coronary calcification is defined as the angiographic ﻿appearance of radiopacities without cardiac motion before contrast injection affecting both sides of the arterial wall (tram-track appearance)^41^. Besides being associated with larger plaque burden, a greater degree of lesion complexity (i.e., involvement of coronary bifurcation or chronic total occlusion [CTO]) and vulnerability (i.e., microcalcifications, calcified nodules), calcified lesions increase PCI complexity and worsen long term results^42,43^.

Lesion preparation may also be hampered by challenges in crossing and dilating lesions using standard devices (i.e., semi-compliant, non-compliant balloon). Of note, inadequate lesion preparation increases the risk ﻿of stent loss, stent under-expansion, stent-malapposition and asymmetric and eccentric lumen enlargement, with higher rates of intraprocedural complication (i.e., no reflow, coronary dissection, or perforation) and long-term adverse events^44^. Adequate lesion preparation is key for good outcomes following PCI, including those performed with DCBs, as poor lesion preparation can lead to less efficient drug transfer to the vessel wall.

Intravascular imaging is pivotal, providing a higher sensitivity for detecting calcium, enabling additional assessment of the calcium’s properties (depth, thickness, length) and morphology (concentric, eccentric, nodular), which are crucial for procedural optimization, and for providing evidence of effective calcium debulking by showing fractures not detectable with angiography^45^.

Considering the circumferential calcium arc, calcified lesions can be divided into eccentric (arc < 180°), concentric (arc > 180°) and nodular (eruptive protrusion into the lumen). Calcium can also be divided into superficial (located at < 50% of the depth of the plaque) or deep (located at > 50%)^46^.

The morphological characteristics of calcium which are associated with sub-optimal results with DES-based PCI include a superficial calcium angle >270°, calcium length> 5 mm, 360° of superficial calcium or a calcified nodule according to IVUS analysis^47^, or an angle >180°, calcium thickness >0.5 mm, and calcium length >5 mm, as per OCT^48^. ﻿To date, there are no RCTs assessing the performance of DCBs in calcified lesions, and moreover angiography-defined severely calcified lesions have traditionally been excluded from DCB RCTs. As obtaining good angiographic results with balloon angioplasty in heavily calcified lesion is challenging and as the drug may be less active in a calcific lesion, data and experience on DCB results in these lesions remains scarce. In the DEBUT RCT, which evaluated a DCB-only approach in HBR patients, 10% of randomized lesions were judged to be calcified and rotational atherectomy was used in 5%^49^. Further registries have suggested the feasibility of a DCB-only approach after rotational atherectomy, while no data are available on this approach after orbital atherectomy or intra-vascular lithotripsy in de novo coronary artery lesions^50,51^.

DCB ARC supports the use of intravascular imaging as an adjunctive technique in dedicated DCB RCTs, with the aim optimal lesion preparation before DCB treatment. DCB ARC will have to document in its electronic case report form (eCRF) which adjunctive devices, such as scoring/cutting balloons, intravascular lithotripsy, and laser have been used for optimal lesion preparation in order to investigate the favorable or unfavorable interaction of DCBs with these adjunctive devices.

- ***High risk lesions (flow limiting/non flow limiting)***

Vulnerable atherosclerotic plaques are at increased risk of destabilization leading to adverse events ^52^. The three main underlying lesion types prone to thrombosis are plaque rupture, plaque erosion and calcified nodules, with the former the commonest cause of ﻿coronary thrombotic events and cardiovascular death.

To date, only the DEBuT-LRP study (NCT04765956) has investigated the role of DCB in the treatment of vulnerable plaques. DCB ARC suggests using intravascular imaging as an adjunctive technique in DCB trials focused on the detection and treatment of vulnerable coronary plaques (**Supplementary** **Table 5**).

- ***Chronic total occlusion***

DCB ARC endorses the definitions and classifications proposed in the CTO ARC^53^. Briefly, ﻿a CTO is considered as an occlusion with the absence of antegrade flow with a documented (definite CTO) or presumed (probable CTO) duration of ≥3 months.

In CTO-dedicated DCB studies two different approaches could be tested: a DCB-only approach that appears to be feasible when wiring (anterograde or retrograde) is intraplaque, and not feasible for dissection re-entry techniques; a blended DCB and DES approach to reduce overall stent length, especially distally where estimation of true vessel size could be challenging.

1. ***Bifurcations***

DCB ARC endorses the definitions and classifications provided in the Bif ARC^54,55^.

Two different types of studies with DCBs in coronary bifurcations can be conceived: the first is a DCB-only strategy, with DCB use in the main vessel across the SB with or without DCB use in the SB (leave nothing behind strategy). Alternatively, DCBs can be used to treat the SB in the setting of a provisional bifurcation technique, either before (if planned) or after (as a bailout if required after main vessel stent) a DES is used in the main branch across the SB (blended strategy).

Some concerns have emerged with the use of DCBs during kissing-balloon inflations, due to the time required and the proximal interaction of the two balloons, which might impact on the delivery of the antiproliferative drug to the vessel wall. A similar concern relates to the use of DCBs to treat the SB after DES implantation in the main branch across the SB, as the interaction between the balloon and stent’s struts, potentially compromises drug delivery.

An approach to a DCB-only bifurcation PCI is comprehensively laid out in the recent international consensus document^36^. However, evidence of the value of a systematic use of DCB in the side-branch in patients with bifurcation lesions treated with provisional stenting strategy is lacking. The ostium of a side-branch may experience acute elastic recoil which is not prevented by DCB. Clinical trials evaluating the performance of DCB in bifurcation lesions are summarized in **Supplementary** **Table 6**, while the technical use of DCB in bifurcation lesions is presented in **Supplementary Figure 3**.

1. ***High bleeding risk***

DCB ARC recommends assessing patients deemed at HBR according to the ﻿ARC-HBR definitions^56^. Briefly, HBR is defined as a 1-year risk of Bleeding ARC (BARC) 3 or 5 bleeding ≥4% or of an intracranial hemorrhage ≥1%. ﻿ARC-HBR proposed twenty clinical criteria, with patients at HBR if at least 1 major or 2 minor criteria are met. DCB ARC highlights the need for investigating whether DCBs allow de-escalation of P2Y12 inhibitors or early discontinuation of DAPT (P2Y12 inhibitor or aspirin discontinuation) in HBR patients.

According to the DEBUT RCT, DCB-only PCI was found to be superior to BMS implantation in patients deemed at HBR in terms of major adverse cardiac events (1.9% vs. 12.4%; p=0.003 for superiority) at 9 months^49^. However, the optimal duration of dual anti-platelet therapy (DAPT) was not investigated in this trial. In a prespecified subgroup analysis of the BASKET-SMALL 2 trial addressing the HBR cohort, a trend towards reduced severe bleedings was seen after DCB-only PCI and a shorter duration of DAPT as compared to DES and standard DAPT^57^.

Whilst the use of current generation DES allows a short 1-month DAPT^58^, the optimal composition and duration of antiplatelet therapy after DCB-only PCI is not yet known. The current consensus on DAPT duration after DCB-only PCI in CCS patients is 1-month, stemming from the first RCT of DCBs for the treatment of ISR^59^, with this duration then adopted for the treatment of all *de novo* lesions^36,60^. Due to the lack of metallic foreign body, use of DCBs could offer advantages for patients at HBR including the shortening of DAPT to less than 1-month or, in case of life-threating bleeding, the possibility of stopping antiplatelet therapy during the first month. Recent registry studies suggest that DCB-only PCI can be safely done using a single anti-platelet in selected populations^61^.

Given the lack of dedicated powered RCTs in HBR patients, comparing DCBs to current generation DES, DCB ARC recognizes the need for further powered and high quality RCTs.

Clinical trials evaluating the performance of DCBs in HBR populations are summarized in **Supplementary** **Table 4**.

**DCB and international guidelines**

**﻿**Guidelines evaluate and summarize available evidence with the aim of assisting health professionals in proposing the best diagnostic or therapeutic approach for an individual patient with a given condition. United States and European guidelines weigh the class of recommendation according to the strength of the available evidence. DCB ARC supports a Class I recommendation to be used in the presence of evidence from superiority RCT that a given treatment is beneficial, useful and effective, preferentially. When evidence comes from non-inferiority RCTs, the same class of recommendation could be used in certain clinical settings, in which the avoidance of permanent implants is particularly advantageous (i.e., ISR). The international community should be aware that in the specific field of DCB, their expected benefits in terms of improved clinical outcomes may not be evident short term, and might require long- and very-long term follow-up time. Therefore, the adoption and advancement of DCB technology is expected to progress further as long-term follow-up data accumulate.

**Supplementary Figure 1. Angiography changes after treatments with DCB or DES (**From Ono M et al. Rationale and Design of the TRANSFORM I Trial. Cardiovasc Revascularization Med. 2021; 25:29–35. <https://doi.org/10.1016/j.carrev.2020.10.004>)


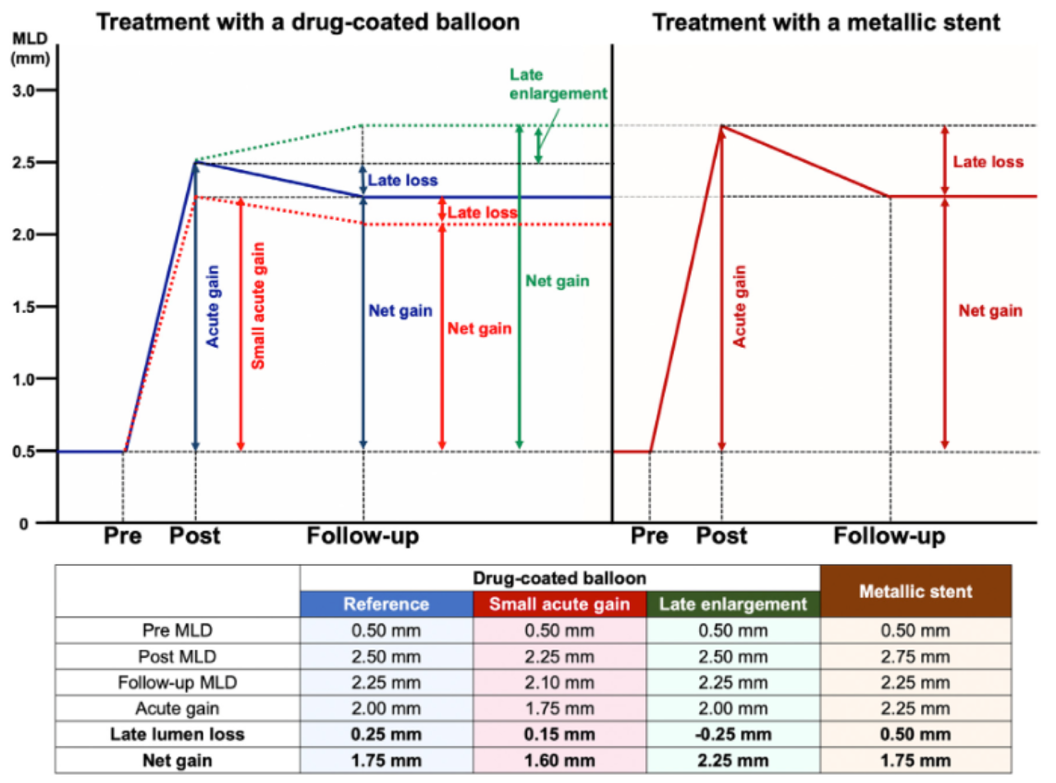


MLD, minimal lumen diameter; mm, millimeter

**Supplementary Figure 2. ﻿Decision tree for statistical models (from Hara H et al. Statistical methods for composite endpoints. *EuroIntervention*. 2021;16:E1484–E1495)**

**
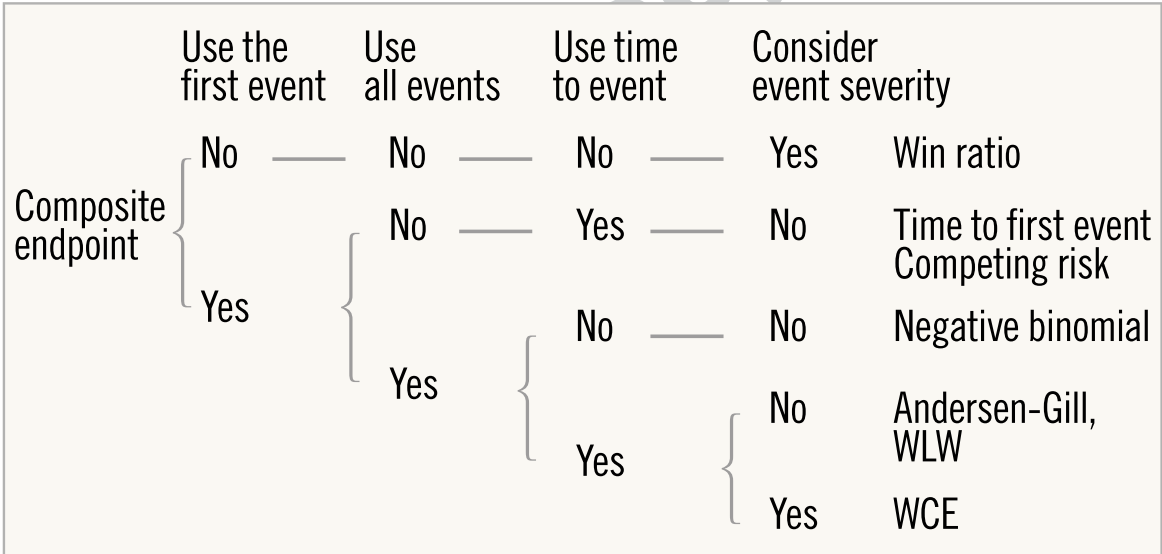
WCE: weighted composite endpoint; WLW: Wei-Lin-Weissfeld**

**Supplementary Figure 3. DCB use in bifurcation lesions**

In cases where disease involves both the MV and the SB (MEDINA 1-1-1 or 0-1-1; top panels), the use of DCB can follow either a “leave nothing behind” strategy, such as DCB treatment across the SB only or DCBs kissing balloon inflation, or a “blended” strategy with DES, with DCB treatment performed either before or after provisional DES implantation.

In cases where the side branch is not diseased (MEDINA x-x-0; bottom panel left), DCB treatment can follow a “leave nothing behind” strategy, such as DCB treatment across the SB only.

In case of side-branch only disease (MEDINA 0-0-1; bottom panel right), DCB treatment can follow a “leave nothing behind” strategy, such as DCB inflation to the SB only.

DCB, drug coated balloon; DES, drug eluting stent; IVL, intravascular Lithotripsy; MV, main-vessel; NC, non-compliant; POT, proximal optimization technique; SB, side-branch; SC, semi-compliant.


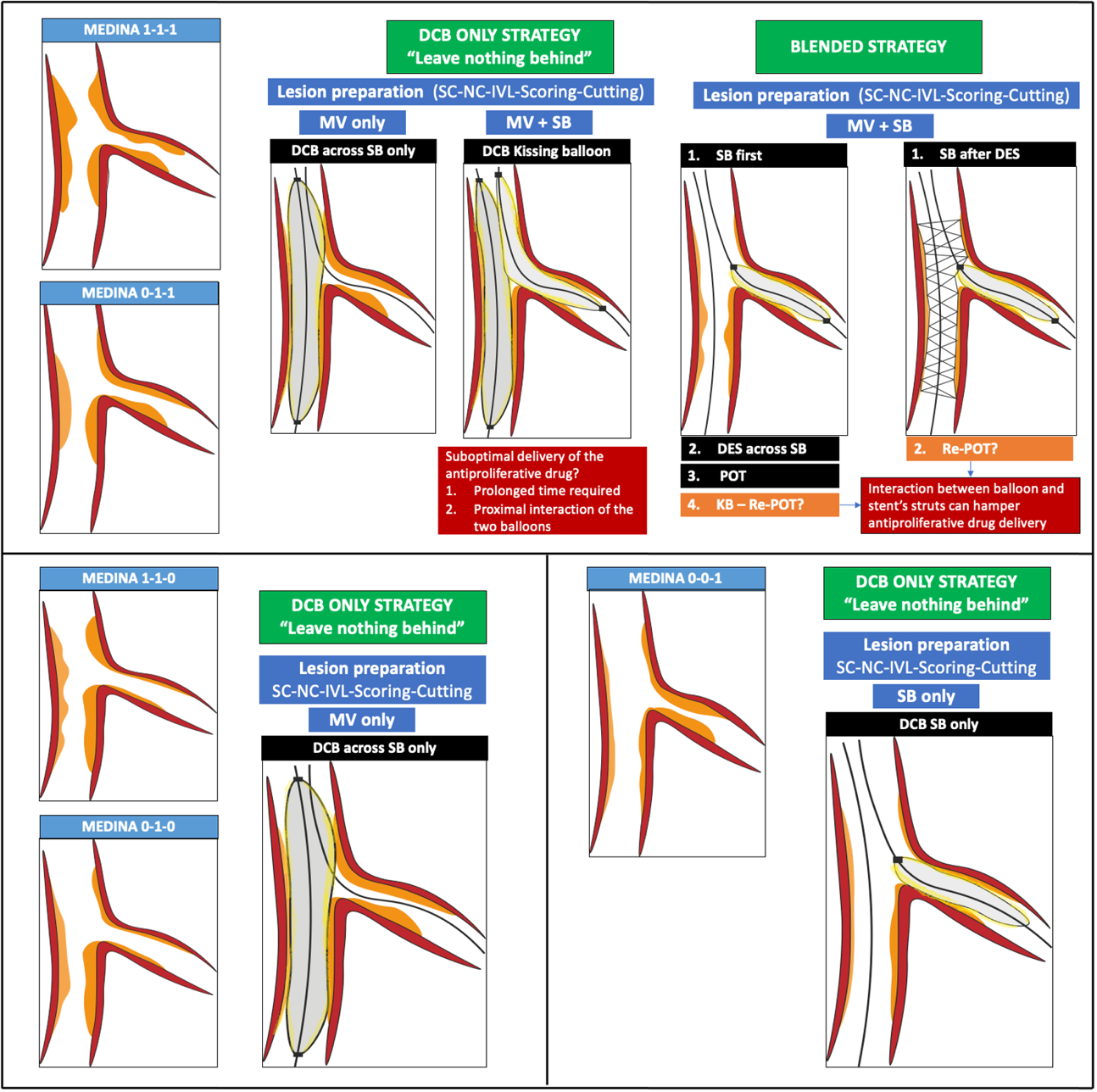


Bibliography

1. Byrne RA, Serruys PW, Baumbach A*, et al.* Report of a European Society of Cardiology-European Association of Percutaneous Cardiovascular Interventions task force on the evaluation of coronary stents in Europe: executive summary. *Eur Heart J* 2015;**36**:2608-2620. doi: 10.1093/eurheartj/ehv203

2. FDA. Investigational Device Exemptions for Early Feasibility Medical Device Clinical Stud- ies, Including Certain First in Human Studies. <https://www.fda.gov/media/81784/download>

3. Muramatsu T, Kozuma K, Tanabe K*, et al.* Clinical expert consensus document on drug-coated balloon for coronary artery disease from the Japanese Association of Cardiovascular Intervention and Therapeutics. *Cardiovasc Interv Ther* 2023;**38**:166-176. doi: 10.1007/s12928-023-00921-2

4. Chen Y, Wang J, Liu B*, et al.* China Expert Consensus on Clinical Application of the Drug‑Coated Balloon. In: Cardiology Plus; 2016.

5. Berkhout C, Berbra O, Favre J*, et al.* Defining and evaluating the Hawthorne effect in primary care, a systematic review and meta-analysis. *Front Med (Lausanne)* 2022;**9**:1033486. doi: 10.3389/fmed.2022.1033486

6. Byrne RA, Capodanno D, Mahfoud F*, et al.* Evaluating the importance of sham-controlled trials in the investigation of medical devices in interventional cardiology. *EuroIntervention* 2018;**14**:708-715. doi: 10.4244/EIJ-D-18-00481

7. Al-Lamee R, Thompson D, Dehbi HM*, et al.* Percutaneous coronary intervention in stable angina (ORBITA): a double-blind, randomised controlled trial. *Lancet* 2018;**391**:31-40. doi: 10.1016/S0140-6736(17)32714-9

8. Ishibashi Y, Muramatsu T, Nakatani S*, et al.* Incidence and Potential Mechanism(s) of Post-Procedural Rise of Cardiac Biomarker in Patients With Coronary Artery Narrowing After Implantation of an Everolimus-Eluting Bioresorbable Vascular Scaffold or Everolimus-Eluting Metallic Stent. *JACC Cardiovasc Interv* 2015;**8**:1053-1063. doi: 10.1016/j.jcin.2015.06.001

9. Généreux P, Stone GW, Harrington RA*, et al.* Impact of intraprocedural stent thrombosis during percutaneous coronary intervention: insights from the CHAMPION PHOENIX Trial (Clinical Trial Comparing Cangrelor to Clopidogrel Standard of Care Therapy in Subjects Who Require Percutaneous Coronary Intervention). *J Am Coll Cardiol* 2014;**63**:619-629. doi: 10.1016/j.jacc.2013.10.022

10. Thygesen K, Alpert JS, Jaffe AS*, et al.* Fourth universal definition of myocardial infarction (2018). *Eur Heart J* 2019;**40**:237-269. doi: 10.1093/eurheartj/ehy462

11. Maron DJ, Hochman JS, Reynolds HR*, et al.* Initial Invasive or Conservative Strategy for Stable Coronary Disease. *N Engl J Med* 2020;**382**:1395-1407. doi: 10.1056/NEJMoa1915922

12. Spertus JV, Hatfield LA, Cohen DJ*, et al.* Integrating Quality of Life and Survival Outcomes in Cardiovascular Clinical Trials. *Circ Cardiovasc Qual Outcomes* 2019;**12**:e005420. doi: 10.1161/CIRCOUTCOMES.118.005420

13. Porter ME, Larsson S, Lee TH. Standardizing Patient Outcomes Measurement. *N Engl J Med* 2016;**374**:504-506. doi: 10.1056/NEJMp1511701

14. Magnuson EA, Chinnakondepalli K, Vilain K*, et al.* Cost-Effectiveness of Percutaneous Coronary Intervention Versus Bypass Surgery for Patients With Left Main Disease: Results From the EXCEL Trial. *Circ Cardiovasc Interv* 2022;**15**:e011981. doi: 10.1161/CIRCINTERVENTIONS.122.011981

15. Brazier JE, Harper R, Jones NM*, et al.* Validating the SF-36 health survey questionnaire: new outcome measure for primary care. *BMJ* 1992;**305**:160-164. doi: 10.1136/bmj.305.6846.160

16. Serruys PW, Hara H, Garg S*, et al.* Coronary Computed Tomographic Angiography for Complete Assessment of Coronary Artery Disease: JACC State-of-the-Art Review. *J Am Coll Cardiol* 2021;**78**:713-736. doi: 10.1016/j.jacc.2021.06.019

17. Yan RT, Miller JM, Rochitte CE*, et al.* Predictors of inaccurate coronary arterial stenosis assessment by CT angiography. *JACC Cardiovasc Imaging* 2013;**6**:963-972. doi: 10.1016/j.jcmg.2013.02.011

18. Collet C, Chevalier B, Cequier A*, et al.* Diagnostic Accuracy of Coronary CT Angiography for the Evaluation of Bioresorbable Vascular Scaffolds. *JACC Cardiovasc Imaging* 2018;**11**:722-732. doi: 10.1016/j.jcmg.2017.04.013

19. Onuma Y, Dudek D, Thuesen L*, et al.* Five-year clinical and functional multislice computed tomography angiographic results after coronary implantation of the fully resorbable polymeric everolimus-eluting scaffold in patients with de novo coronary artery disease: the ABSORB cohort A trial. *JACC Cardiovasc Interv* 2013;**6**:999-1009. doi: 10.1016/j.jcin.2013.05.017

20. Weinstein JN, Lurie JD, Tosteson TD*, et al.* Surgical versus nonsurgical treatment for lumbar degenerative spondylolisthesis. *N Engl J Med* 2007;**356**:2257-2270. doi: 10.1056/NEJMoa070302

21. Stone GW, Maehara A, Lansky AJ*, et al.* A prospective natural-history study of coronary atherosclerosis. *N Engl J Med* 2011;**364**:226-235. doi: 10.1056/NEJMoa1002358

22. Redfors B, Gregson J, Crowley A*, et al.* The win ratio approach for composite endpoints: practical guidance based on previous experience. *Eur Heart J* 2020;**41**:4391-4399. doi: 10.1093/eurheartj/ehaa665

23. Hara H, Onuma Y, Serruys PW. Reply: Composite endpoints in clinical trials - simplicity or perfection? *EuroIntervention* 2022;**17**:1121-1122. doi: 10.4244/EIJ-D-21-00440R

24. Tang Y, Fitzpatrick R. Sample size calculation for the Andersen-Gill model comparing rates of recurrent events. *Stat Med* 2019;**38**:4819-4827. doi: 10.1002/sim.8335

25. Bakal JA, Westerhout CM, Armstrong PW. Impact of weighted composite compared to traditional composite endpoints for the design of randomized controlled trials. *Stat Methods Med Res* 2015;**24**:980-988. doi: 10.1177/0962280211436004

26. Giacoppo D, Alfonso F, Xu B*, et al.* Drug-Coated Balloon Angioplasty Versus Drug-Eluting Stent Implantation in Patients With Coronary Stent Restenosis. *J Am Coll Cardiol* 2020;**75**:2664-2678. doi: 10.1016/j.jacc.2020.04.006

27. Alfonso F, Coughlan JJ, Giacoppo D, Kastrati A, Byrne RA. Management of in-stent restenosis. *EuroIntervention* 2022;**18**:e103-e123. doi: 10.4244/EIJ-D-21-01034

28. Alfonso F, Cuesta J. A novel clinical score to predict repeat coronary interventions in patients with drug-eluting stent restenosis. *EuroIntervention* 2023;**18**:e1297-e1299. doi: 10.4244/EIJ-E-23-00006

29. Alfonso F, Byrne RA, Rivero F, Kastrati A. Current treatment of in-stent restenosis. *J Am Coll Cardiol* 2014;**63**:2659-2673. doi: 10.1016/j.jacc.2014.02.545

30. Alfonso F, Cequier A, Angel J*, et al.* Value of the American College of Cardiology/American Heart Association angiographic classification of coronary lesion morphology in patients with in-stent restenosis. Insights from the Restenosis Intra-stent Balloon angioplasty versus elective Stenting (RIBS) randomized trial. *Am Heart J* 2006;**151**:681.e681-681.e689. doi: 10.1016/j.ahj.2005.10.014

31. Mehran R, Dangas G, Abizaid AS*, et al.* Angiographic patterns of in-stent restenosis: classification and implications for long-term outcome. *Circulation* 1999;**100**:1872-1878. doi:

32. Solinas E, Dangas G, Kirtane AJ*, et al.* Angiographic patterns of drug-eluting stent restenosis and one-year outcomes after treatment with repeated percutaneous coronary intervention. *Am J Cardiol* 2008;**102**:311-315. doi: 10.1016/j.amjcard.2008.03.060

33. Latib A, Mussardo M, Ielasi A*, et al.* Long-term outcomes after the percutaneous treatment of drug-eluting stent restenosis. *JACC Cardiovasc Interv* 2011;**4**:155-164. doi: 10.1016/j.jcin.2010.09.027

34. Neumann FJ, Sousa-Uva M, Ahlsson A*, et al.* 2018 ESC/EACTS Guidelines on myocardial revascularization. *Eur Heart J* 2019;**40**:87-165. doi: 10.1093/eurheartj/ehy394

35. Jeger RV, Farah A, Ohlow MA*, et al.* Long-term efficacy and safety of drug-coated balloons versus drug-eluting stents for small coronary artery disease (BASKET-SMALL 2): 3-year follow-up of a randomised, non-inferiority trial. *Lancet* 2020;**396**:1504-1510. doi: 10.1016/S0140-6736(20)32173-5

36. Jeger RV, Eccleshall S, Wan Ahmad WA*, et al.* Drug-Coated Balloons for Coronary Artery Disease: Third Report of the International DCB Consensus Group. *JACC Cardiovasc Interv* 2020;**13**:1391-1402. doi: 10.1016/j.jcin.2020.02.043

37. Sanz-Sánchez J, Chiarito M, Gill G*, et al.* Small Vessel Coronary Artery Disease: Rationale for Standardized Definition and Critical Appraisal of the Literature. *Journal of the Society for Cardiovascular Angiography & Interventions* 2022;**1**. doi: <https://doi.org/10.1016/j.jscai.2022.100403>

38. Ono M, Kawashima H, Hara H*, et al.* A Prospective Multicenter Randomized Trial to Assess the Effectiveness of the MagicTouch Sirolimus-Coated Balloon in Small Vessels: Rationale and Design of the TRANSFORM I Trial. *Cardiovasc Revasc Med* 2021;**25**:29-35. doi: 10.1016/j.carrev.2020.10.004

39. Madhavan MV, Kirtane AJ, Redfors B*, et al.* Stent-Related Adverse Events >1 Year After Percutaneous Coronary Intervention. *J Am Coll Cardiol* 2020;**75**:590-604. doi: 10.1016/j.jacc.2019.11.058

40. Scarsini R, Fezzi S, Leone AM*, et al.* Functional Patterns of Coronary Disease: Diffuse, Focal, and Serial Lesions. *JACC Cardiovasc Interv* 2022;**15**:2174-2191. doi: 10.1016/j.jcin.2022.07.015

41. De Maria GL, Scarsini R, Banning AP. Management of Calcific Coronary Artery Lesions: Is it Time to Change Our Interventional Therapeutic Approach? *JACC Cardiovasc Interv* 2019;**12**:1465-1478. doi: 10.1016/j.jcin.2019.03.038

42. Onuma Y, Tanimoto S, Ruygrok P*, et al.* Efficacy of everolimus eluting stent implantation in patients with calcified coronary culprit lesions: two-year angiographic and three-year clinical results from the SPIRIT II study. *Catheter Cardiovasc Interv* 2010;**76**:634-642. doi: 10.1002/ccd.22541

43. Bourantas CV, Zhang YJ, Garg S*, et al.* Prognostic implications of coronary calcification in patients with obstructive coronary artery disease treated by percutaneous coronary intervention: a patient-level pooled analysis of 7 contemporary stent trials. *Heart* 2014;**100**:1158-1164. doi: 10.1136/heartjnl-2013-305180

44. Kawashima H, Serruys PW, Hara H*, et al.* 10-Year All-Cause Mortality Following Percutaneous or Surgical Revascularization in Patients With Heavy Calcification. *JACC Cardiovasc Interv* 2022;**15**:193-204. doi: 10.1016/j.jcin.2021.10.026

45. Araki M, Park SJ, Dauerman HL*, et al.* Optical coherence tomography in coronary atherosclerosis assessment and intervention. *Nat Rev Cardiol* 2022;**19**:684-703. doi: 10.1038/s41569-022-00687-9

46. Mintz GS, Nissen SE, Anderson WD*, et al.* American College of Cardiology Clinical Expert Consensus Document on Standards for Acquisition, Measurement and Reporting of Intravascular Ultrasound Studies (IVUS). A report of the American College of Cardiology Task Force on Clinical Expert Consensus Documents. *J Am Coll Cardiol* 2001;**37**:1478-1492. doi: S0735109701011755 [pii]

47. Zhang M, Matsumura M, Usui E*, et al.* Intravascular Ultrasound-Derived Calcium Score to Predict Stent Expansion in Severely Calcified Lesions. *Circ Cardiovasc Interv* 2021;**14**:e010296. doi: 10.1161/CIRCINTERVENTIONS.120.010296

48. Fujino A, Mintz GS, Matsumura M*, et al.* A new optical coherence tomography-based calcium scoring system to predict stent underexpansion. *EuroIntervention* 2018;**13**:e2182-e2189. doi: 10.4244/EIJ-D-17-00962

49. Rissanen TT, Uskela S, Eränen J*, et al.* Drug-coated balloon for treatment of de-novo coronary artery lesions in patients with high bleeding risk (DEBUT): a single-blind, randomised, non-inferiority trial. *Lancet* 2019;**394**:230-239. doi: 10.1016/S0140-6736(19)31126-2

50. Rissanen TT, Uskela S, Siljander A*, et al.* Percutaneous Coronary Intervention of Complex Calcified Lesions With Drug-Coated Balloon After Rotational Atherectomy. *J Interv Cardiol* 2017;**30**:139-146. doi: 10.1111/joic.12366

51. Dong H, Shan Y, Gong S*, et al.* Clinical research of drug-coated balloon after rotational atherectomy for severe coronary artery calcification. *BMC Cardiovasc Disord* 2023;**23**:40. doi: 10.1186/s12872-023-03071-8

52. Muller JE, Tofler GH, Stone PH. Circadian variation and triggers of onset of acute cardiovascular disease. *Circulation* 1989;**79**:733-743. doi: 10.1161/01.cir.79.4.733

53. Ybarra LF, Rinfret S, Brilakis ES*, et al.* Definitions and Clinical Trial Design Principles for Coronary Artery Chronic Total Occlusion Therapies: CTO-ARC Consensus Recommendations. *Circulation* 2021;**143**:479-500. doi: 10.1161/CIRCULATIONAHA.120.046754

54. Lunardi M, Louvard Y, Lefèvre T*, et al.* Definitions and Standardized Endpoints for Treatment of Coronary Bifurcations. *EuroIntervention* 2022. doi: 10.4244/EIJ-E-22-00018

55. Lunardi M, Louvard Y, Lefèvre T*, et al.* Definitions and Standardized Endpoints for Treatment of Coronary Bifurcations. *J Am Coll Cardiol* 2022;**80**:63-88. doi: 10.1016/j.jacc.2022.04.024

56. Urban P, Mehran R, Colleran R*, et al.* Defining high bleeding risk in patients undergoing percutaneous coronary intervention: a consensus document from the Academic Research Consortium for High Bleeding Risk. *Eur Heart J* 2019;**40**:2632-2653. doi: 10.1093/eurheartj/ehz372

57. Scheller B, Rissanen TT, Farah A*, et al.* Drug-Coated Balloon for Small Coronary Artery Disease in Patients With and Without High-Bleeding Risk in the BASKET-SMALL 2 Trial. *Circ Cardiovasc Interv* 2022;**15**:e011569. doi: 10.1161/CIRCINTERVENTIONS.121.011569

58. Valgimigli M, Frigoli E, Heg D*, et al.* Dual Antiplatelet Therapy after PCI in Patients at High Bleeding Risk. *N Engl J Med* 2021;**385**:1643-1655. doi: 10.1056/NEJMoa2108749

59. Scheller B, Hehrlein C, Bocksch W*, et al.* Treatment of coronary in-stent restenosis with a paclitaxel-coated balloon catheter. *N Engl J Med* 2006;**355**:2113-2124. doi: NEJMoa061254 [pii]

10.1056/NEJMoa061254

60. Corballis NH, Wickramarachchi U, Vassiliou VS, Eccleshall SC. Duration of dual antiplatelet therapy in elective drug-coated balloon angioplasty. *Catheter Cardiovasc Interv* 2020;**96**:1016-1020. doi: 10.1002/ccd.28632

61. Räsänen A, Kärkkäinen JM, Eranti A, Eränen J, Rissanen TT. Percutaneous coronary intervention with drug-coated balloon-only strategy combined with single antiplatelet treatment in patients at high bleeding risk: Single center experience of a novel concept. *Catheter Cardiovasc Interv* 2023. doi: 10.1002/ccd.30558

**Supplementary Table 1. Clinical trials evaluating the performance of DCBs in in-stent restenosis**

| **BMS ISR** | | | | | | | | | | | | | |
| --- | --- | --- | --- | --- | --- | --- | --- | --- | --- | --- | --- | --- | --- |
| **Study name** | **Design** | **DCB** | **Comparator** | **n** | **ISR** | **Inclusion** | **Exclusion** | **Reference vessel** | **Primary endpoint** | **Secondary endpoints** | **Angiographic follow-up (p value)** | **MACE (p value)** | **TLR (p value)** |
| **PACCOCATH ISR I -II** | 1:1 RCT  Open-label  Corelab  CEC | PCB | POBA | 108 | BMS (96)  DES (4) | CCS or UA or ischaemia | MI<72H, CKD (crea>2), allergies  Thrombus, severe calcification, stenosis<70% | RVD>2.5, length<30mm | 6M LLL | 6M ISR  12-24-60M ST, TLR, MI, stroke, death | 6M  LLL 0.14 ± 0.46 mm vs 0.81 ±0.79  (0.001) | 12M  9 vs 44 (0.001)  60M  27.8 vs 59.3 (0.009) | 12M  4 vs 37 (0.001)  60M  9.3 vs 38.9 (0.004) |
| **PEPCAD II** | 1:1 RCT  Open-label  Corelab  CEC | PCB  Paccocath | PES | 131 | BMS | CCS or UA or ischaemia | MI<48H, GFR<30, Allergies, life expectancy <2Y  DS<70%, LM, stents covering a major side branch >2mm | RVD 2.5-3.5, length ≤22mm | 6M LLL | 6M ISR  12-36M MACE (ST, TLR, MI, death) | 6M  LLL 0.17 ± 0.42 mm vs 0.38 ±0.61  (0.03) | 12M  9 vs 22 (0.08)  36M  34.8 vs 41.5 (?) | 12M  6 vs 15 (0.015)  36M  - |
| **RIBS V** | 1:1 RCT  Open-label  Corelab  CEC | PCB  Sequent please | EES | 189 | BMS | CCS or ischaemia  DS>50% | Small vessels (<2mm), diffuse lesions (>30mm)  Early (<1m) ISR, MI, thrombus | RVD>2mm, length<30mm | 9M in-segment MLD | 12-36M MACE, ISR | 6M  LLL 0.14 ± 0.5 mm vs 0.04 ±0.5 (0.14)  ISR (9.5 vs 4.7) (0.22) | 12M  8 vs 6 (0.60)  36M  12 vs 10 (0.64) | 12M  6 vs 1 (0.09)  36M  8 vs 2 (0.04) |
| **SEDUCE** | 1:1 RCT  Open-label  Corelab  CEC | PCB  Sequent please | EES | 50 | BMS | Any ISR | LVEF <30, crea>2, LM, bifurcations, LE<1Y | RVD 2-4mm, length<24mm | 9M uncovered struts (OCT) | 9M LLL  12M MACE, TLR | 6M  1.4 vs 3.1% (0.025)  LLL 0.28 vs 0.07 (0.1) | 12M  - | 12M  4.2 vs 8 (0.576) |
| **TIS** | 1:1 RCT  Open-label  Corelab  CEC | PCB  Sequent please | EES | 136 | BMS | Any ISR (DS>50) | LE<1Y, contraindication to DAPT | Any | 12M LLL | 12-36M MACE TVR | 6M  LLL 0.02 vs 0.19 (0.0004)  ISR ﻿8.7 vs 19.12% (P=0.078) | 12M  10.3 vs 19.1 (0.213)  36M  19.1 vs 29.4 (0.230) | 12M  7.6 vs 16.2 (0.110)  36M  12.9 vs 22.2 (0.205) |
| **DES ISR** | | | | | | | | | | | | | |
| **PEPCAD DES, 2012** | 1:1 RCT  Open-label  Corelab  CEC | PCB  Sequent please | POBA | 110 | DES | Any ISR | Thrombus, bifurcation, grafts, CTO, ostial, LM, planned surgery | RVD 2.5-3.5; length <22mm | 6M LLL | 6-36M MACE or TLR | 6M  LLL 0.43 ± 0.61 vs 1.03 ± 0.77 mm  ISR 17.2 vs 58.1% (0.001) | 6M  16.7 vs 50 (0.001)  36M  20.8 vs 52.6 (0.001) | 6M  15.3 vs 36.8 (0.005)  36M  19.4 vs 36.8 (0.046) |
| **PEPCAD CHINA ISR, 2014** | 1:1 RCT  Open-label  Corelab  CEC | PCB  Sequent please | PES | 220 | DES | ISR DS>70 or 50 with ischemia | MI<7D, bifurcation Sb >2.5, thrombus, NYHA IV, severe VHD, stroke<6M, GFR<30 | RVD 2.5-4  Length <30mm | 9M LLL (non-inferiority) | 9M ISR  12-24M TLF, TLR | 9M  LLL 0.46 ± 0.51 vs 0.55 ± 0.61 mm (0.0005)  ISR 13 vs 10 (0.16) | 12M  TLF 16.5 vs 16 (0.92)  24M  TLF 16.8 vs 18.6 (0.73) | 12M  15.6 vs 12.3 (0.48)  24M  15.9 vs 13.7 (0.66) |
| **ISAR DESIRE III, 2013** | 1:1 RCT  Open-label  Corelab  CEC | PCB  Sequent please | PES vs POBA | 402 | DES | ISR>50% | STEMI<48H, grafts, LM, bifurcation, GFR<30, shock, LE<12M, allergy | Any | 6-8M DS (non-inferiority) | 12-36M TLR, DEATH+MI, St | 6-8M  DS 38 vs 37.4% (0.007) | 12M  23.5 vs 19.3 (0.5) vs 46 (0.001)  36M  38 vs 38 (0.91) vs 56 (0.001) | 12M  22 vs 13 (0.09) vs 43 (0.001)  36M  33 vs 24 (0.11) vs 51 (0.001) |
| **ISAR DESIRE IV, 2017** | 1:1 RCT  Open-label  Corelab  CEC | PCB  Pantera lux | Scoring vs POBA | 252 | DES | ISR>50% | LM, MI<48H, LE<12M, GFR<30 | Any | 6-8M DS (non-inferiority) | 6-8M ISR  12M death MI TLR ST | 6-8M  DS 35 ±17 vs 40 ± 21 (0.047)  LLL 0.31 ± 0.59 vs 0.41 ± 0.74 mm (0.27)  ISR 19 vs 32 (0.026) | 12M  18.4 vs 23.3 (0.35) | 12M  16.2 vs 21.8 (0.26) |
| **RIBS IV, 2015** | 1:1 RCT  Open-label  Corelab  CEC | PCB | EES | 309 | DES | ISR | CTO, early<1M ISR, acyte MI, thrombus, multiple TLR, LE<1Y | RVD>2.0 mm length<30nn | 6-9M In-segment MLD (superiority of EES) | 12-36M MACE, TLR | 6-9M  MLD 1.80±0.6 vs 2.03±0.7 (0.004)  ISR 19 vs 11% (0.06) | 12M  18 vs 10 (0.04)  36M  20.1 vs 12.3 (0.04) | 12M  16 vs 8 (0.035)  36M  15.6 vs 7 (0.015) |
| **RESTORE, 2018** | 1:1 RCT  Open-label  Corelab  CEC | PCB  ﻿SeQuent Please | EES  Xience | 172 | DES | DES ISR DS>50% | LE<1Y, contraindication to paclitaxel/everolimus, DAPT | Any | 9M LLL (superiority of DCB) | 9M ﻿MLD and DS  12M MACE, TLR | 9M  LLL 0.15 ± 0.49 vs 0.19 ± 0.41 (0.54) | 12M  7.0 vs 4.7 (0.51) | 12M  5.8 vs 1.2 (0.10) |
| **FILM LIMUS, 2019** | 1:1 RCT  Open-label  Corelab  CEC | SCB  ﻿SeQuent Neo | PCB  ﻿SeQuent Please Neo 3 | 50 | ISR | CCS or UA  DES ISR up to 2 lesions | MI<72H, crea>2, contraindications to DAPT, paclitaxel, sirolimus | Length<35mm  RVD <2.5mm | 6M LLL | Procedural success (<30% final stenosis, TMI 3, no flow limiting dissections)  6-12M MACE, CD, St, TLR, ISR | 6M  LLL 0.21 ± 0.54 vs 0.17 ± 0.55 (0.794) | 12M  16 vs 12 (0.99) | 12M  16 vs 12 (0.99) |
| **PREVAIL** | Open label  Single arm  ISR, De novo, small vessels | Prevail | - | 50 | ? | Any De novo small or ISR | PCI of target vessel<9M, stroke TIA <&M, MI<72H | Length<25mm, RVD 2-4mm, DS 50-100 | 6M LLL | 6M DS, MLD, ISR  1M, 6M, 12M death, MI, MACE, TLR, TVF, TLF | LLL 0.05﻿±0.44  ISR 10% | 6% | 6% |
| **AGENT ISR, 2021** | 1:1 RCT  Open-label non-inferiority  Corelab  CEC | Agent  PCB | Sequent please PCB | 125 | Any | Any | LM, recent or planned PCI, CTO, recent MI | Length<28mm  RVD 2-3.5 | 6M LLL | 1-6-12M MI, death, TLR, TVR | 6M  LLL ﻿0.397±0.43 vs ﻿0.393±0.536 mm (p non inferiority 0.046) | - | 12M  7.7 vs 10 (0.89) |
| **RESTORE ISR China, 2018** | 1:1RCT  Open-label non-inferiority  Corelab  CEC | Restore  PCB (﻿SAFEPAX shellac- ammonium salt excipient) | Sequent please PCB | 240 | Any | Any ISR (Mehran I-III)  DS>70% or 50% with ischemia | MI<7D, >2lesions requiring PCI, bifurcation with SB ≥2.5, thrombus  NYHA IV, stroke<6M, GFR<30, SVHD | RVD 2.5-4 | 9M LLL | Acute success (device, procedure, lesion)  9M ISR  12M TLF, POCE | 9M  LLL ﻿0.38 ±0.50 vs 0.35± 0.47 (p non inferiority 0.02)  ISR 24.6 vs 18.8 (0.29) | 12M  TLF 13.3 vs 12.6 (0.87) | 12M  13.3 vs 11.8 (0.71) |
| **PEPPER trial** | First in man, observational, single arm | Pantera lux PCB | - | 81 | Any | Any | MI<72H, LVEF<30%, allergies | Any | 6M LLL | 6,12M MACE | 6M  LLL 0.07±0.31 mm | 12M  11.8% | 12M  9.2% |
| **GENOSS, 2022** | 1:1RCT  Open-label non-inferiority  Corelab  CEC | Genoss PCB (Shellac + vit E) | Sequent Please | 82 | Any | Mehran I-III DS>50%, More than 90D after stent placement | Acute MI, thrombosis, grafts, Mehran IV, allergies | Any | 6M LLL | 6M MACE, TLR  Device, Lesion, Procedure Success | 6M  LLL 0.15﻿± 0.43 vs 0.24﻿± 0.39 (p non inferiority 0.001) | 6M  7.7 vs 10.3 (0.692) | 6M  5.1 vs 5.1 |
| **BIOLUX, 2018** | 2:1RCT  Open-label non-inferiority  Corelab  CEC | Pantera Lux PCB | SES | 229 | BMS (37) DES (63) | CCS or ischaemia with ISR >50%  In case or two lesions, both need to be treated with DCB | STEMI<72H, LVEF<30, LM, thrombus  Allergies, crea>2.5, LE<18M | RVD<2 />4mm  Length <6 >28mm | 6M LLL | 6M DS, MLD  12M TLF, St  Device success | 6M  LLL 0.03 ± 0.40 vs 0.20±0.70 (0.40) | 12M  TLF  16.9 vs 14.2 (0.65) | 12M  12.5 vs 10.1 (0.82) |
| **DARE, 2018** | 1:1RCT  Open-label non-inferiority  Corelab  CEC | Sequent Please PCB (if crossover BMS in PCB arm) | EES (Xience) | 278 | BMS (44) vs DES (56) | All ISR (>50%) even CTO, ostial, LM, bifurcation, grafts | STEMI, BRS | RVD 2 -4 | 6M MLD | 6M ISR, persisting dissection  MI, TLR; St | 6M  MLD 1.71 ± 0.51 vs 1.74± 0.61 (non inferiority <0.0001) | 12M  10.9 vs 9.2 (0.66) | 12M TVR  7.1 vs 8.8 (0.65) |

BMS, bare metal stent; BRS, bioresorbable scaffold; CEC, central clinical events committee; CCS, chronic coronary syndrome; CTO, chronic total occlusion; DAPT, dual antiplatelet therapy; DCB, drug coated balloon; DES, drug eluting stent; DS, degree of stenosis; EES, everolimus eluting stent; GFR, glomerular filtrate rate; ISR, in-stent restenosis; LLL, late lumen loss; LM, left main; LVEF, left ventricle ejection fraction; M, months; MACE, major adverse cardiac death; MI, myocardial infarction; MLD, minimal lumen diameter; NYHA, New York Heart Association; PCB, paclitaxel coated balloon; PCI, percutaneous coronary intervention; POBA, plain old balloon angioplasty; POCE, patient oriented composite endpoint; RCT, randomized clinical trial; RVD, reference vessel diameter; SES, sirolimus eluting stent; St, stent thrombosis; STEMI, ST-segment elevation MI; TIA, transient ischemic attack; TLF, target lesion failure; TLR, target lesion revascularization; TVR, target vessel revascularization; UA, unstable angina; VHD, valvular heart disease.

**Supplementary Table 2. ISR lesions classification based on angiography, intravascular ultrasound and optical coherence tomography**

| **Angiography Mehran’s classification** | | |
| --- | --- | --- |
| **Class I** | **Focal ISR** | Lesions ≤10 mm in length   - IA: At the un-scaffolded segment (i.e., articulation or gap) - IB: at the proximal or distal margin (but not both) - IC: at the body ﻿of the stent - ID: combination of these sites (multifocal ISR) |
| **Class II** | **Diffuse intrastent** | Lesions >10 mm in length and extended beyond the margin(s) of the stent(s) |
| **Class III** | **Diffuse proliferative** | Lesions >10 mm in length and extended beyond the margin(s) of the stent(s) |
| **Class IV** | **Total occlusion** | Lesions have a TIMI flow grade of 0. |
| **Intravascular ultrasound ISR pattern classification** | | |
| **Class I** | **Focal ISR** | Lumen area <4 mm^2^ and ≤10 mm in length   - Focal body type: confined to the body of stent - Focal marginal type: extending to the margins of stent |
| **Class II** | **Multifocal ISR** | - Multifocal body type: multiple focal ISR lesions confined to the body of the stent without involvement of the stent margins - Multifocal marginal type: multiple focal ISR lesions that included marginal involvement |
| **Class III** | **Diffuse ISR** | Lumen area <4 mm^2^ and >10 mm in length   - Diffuse body type: confined to the body of stent - Diffuse marginal type: extending to the margins of the stent |
| **Optical coherence tomography Waksman’s ISR classification** | | |
| **Type I** | **Mechanical** | - IA: DES under-expansion - IB: DES fracture |
| **Type II** | **Biologic** | - IIA: intimal hyperplasia - IIB: non-calcified neoatherosclerosis - IIC: calcified atherosclerosis |
| **Type III** | **Mixed** | Combination of mechanical and biological mechanisms |
| **Type IV** | **Chronic total occlusion** |  |
| **Type V** | **Multiple stent layers** | More than two metallic layers of stents are present |

DES, drug eluting stent; ISR, in-stent restenosis; TIMI, Thrombolysis in Myocardial Infarction

**Supplementary Table 3. Clinical trials evaluating the performance of DCBs in de-novo small vessels**

| **Study name** | **Design** | **DCB** | **Comparator** | **n** | **Inclusion** | **Exclusion** | **Predilatation/Bail-out stenting** | **Reference vessel** | **Primary endpoint** | **Secondary endpoints** | **Angiographic follow-up (p value)** | **MACE (p value)** | **TLR (p value)** |
| --- | --- | --- | --- | --- | --- | --- | --- | --- | --- | --- | --- | --- | --- |
| **PICCOLETO, 2010** | Single center prospective  1:1 RCT  Autonomous QCA | DIOR PCB | TAXUS Libertè PES | 57 | Predilatation mandatory only in DES group | MI<48H, unstable, crea>2, allergies, LE<2Y | 25 vs 86%  36% | <2.75 | ITT  6M DS  in-segment | 6M MLD, ISR  9M MACE, TLR  Procedural/device success | 6M  DS%43.6±27.4 vs 24.3±25.1 (0.029)  MLD 1.11±0.65 vs 1.94±0.72 (0.0002) | 9M  35.7 vs 13.8 (0.054) | 9M  32.1 vs 10.3 (0.15) |
| **BELLO, 2012** | 1:1 RCT  Open-label non-inferiority  Corelab  CEC | INPACT FALCON PCB | TAXUS Libertè PES | 182 | CCS or UA | MI<48H, previous PCI<3M, LVEF<30%, crea>2, allergies, stroke<6M  3VD, ostial, restenosis, grafts, CTO, thrombus, bifurcation (2Stent, SB﻿≥2.5) | 97 vs 83%  20% | <2.8 | 6M LLL  in-segment | 6M ISR  12-36M MACE, TLR | 6M  LLL 0.08 ± 0.38 vs 0.29±0.44 (0.001)  ISR 9 vs 14 (0.25) | 12M  10 vs 16 (0.21)  36M  14 vs 30 (0.015) | 12M  4.4 vs 7.6 (0.37)  36M  6 vs 13 (0.14) |
| **RESTORE SVD, 2018, 2020** | 1:1 RCT  Open-label non-inferiority  Corelab  CEC | Restore PCB | Resolute Integrity ZES | 230 (32 in very small) | DS>70% or >50% with ischemia | MI<7D, LVEF<35%, CTO, bifurcation, LM, >=2lesions | 100 vs 100  5.2% | 2.25-2.75  Very small 2-2.25  Length<26 | 9M DS (ITT)  In-segment | 9M DS  9M LLL  12-24M TLF | 9M  DS 29.6±2.0 vs 24±2 (non-inferiority 0.001)  LLL 0.26±0.42 vs 0.30±0.35 (0.41) | 12M  9.6 vs 9.6 (1.0)  24M  - | 12M TLF  4.4 vs 2.6 (0.72)  24M  5.2 vs 2.8 (0.5) |
| **BASKET-SMALL2, 2018,2020** | 1:1 RCT  Open-label non-inferiority  Corelab  CEC | Sequent Please PCB | TAXUS PES and XIENCE EES | 758 | CCS, ACS  Successful pre-dilatation | Concomitant PCI in a large (>3mm) on same epicardial artery  Restenosis, LE<1Y, pregnancy | 100 vs 100  5% | RVD 2-3 | 12M MACE (non inferiority) | 12-36M adverse events, clinical benefit | 6M  LLL 0.13 (-0.14 to 0.57) vs 0.10 (-0.16 to 0.34) (0.72) | 12M  8 vs 8 (0.918; non-inferiority 0.015)  36M  15 vs 15 (ns) | 12M  3.4 vs 4.5 (0.438)  36M  9 vs 9 (ns) |
| **BIORISE CHINA, 2022** | 1:1 RCT  Open-label, superiority  Corelab  CEC | BA9 BCB | POBA | 212 | CCS or UA (DS>70 or >50 with ischemia) | MI<1M, severe HF, shock, LVEF<35, allergies, LE<1Y, severe CKD, stroke, GI bleeding<6M, severe liver failure  Thrombus, >=2 non target lesions, CTO, ISR, severe calcification | 100 vs 100  2.8% | RVD 2-2.75  Length≤25 | PP  9M LLL  In-segment | 9M ISR, device, lesion, procedure-success  9-12M MACE, TLR, TLF, POCE, St | 9M  LLL 0.16±0.29 vs 0.30±0.35 (0.001)  Positive remodeling 29 vs 9 %(0.007) | 12M TLF  6.7 vs 13.9 (0.088) | 12M  5.7 vs 10.9 (0.177) |
| **PICCOLETO II, 2022** | 1:1 RCT  Open-label, non-inferiority  Corelab  CEC | Elutax SV/Emperor PCB (dextran) | Xience EES | 232 | CCS or ACS, DS>70% | LE<12M, MI<72H, LVEF<30%, GFR<30  Ostial, LM, CTO, tortuosity, severe calcification, thrombus | 84 vs 69%  6.7% | RVD 2-2.75  Length≤25 | 6M LLL  In-segment | 6M MLD, DS  12-36M MACE | 6M  LLL 0.04 vs 0.17 (non inferiority 0.001; superiority 0.03) | 12M  5.6 vs 7.5 (0.55)  36M  10.8 vs 20.8 (0.046) | 12M  5.6 vs 5.6 (0.80)  36M  8.8 vs 14.8 (0.18) |
| **PEPCAD I, 2010** | Prospective, observational multi-center trial | Sequent please | - | 118 | UA or CCS, single de-novo | MI<48H, GFR<30, allergies, LE<2Y | 30% | 2.25-2.8 | 6M LLL | 6M ISR  12M MACE, TLR | 6M ﻿  LLL 0.28 ± 0.53  ISR 17% | 12M  15% | 12M  12% |

Same as Table 2.

**Supplementary Table 4. Clinical trials evaluating the performance of DCBs in large vessels, myocardial infarction and high bleeding risk patients**

| **LARGE VESSEL DISEASE** | | | | | | | | | | | | | |
| --- | --- | --- | --- | --- | --- | --- | --- | --- | --- | --- | --- | --- | --- |
| **Study name** | Design | DCB | Comparator | n | Inclusion | Exclusion | Predilatation | Reference vessel | Primary endpoint | Secondary endpoints | Angiographic follow-up (p value) | MACE (p value) | TLR (p value) |
| **﻿SCBDNMAL**  **NCT04017364** | 1:1 RCT  Open-label non-inferiority  Corelab  CEC | Sequent SCB | Sequent please PCB | 70 | De novo, CCS or UA (﻿≥70% or ≥50% with ischemia) | MI<72H, allergies, LVEF<30%, RVD<2.5 | Scoring recommended | RVD>2.5 | 6M LLL ITT | Procedural success  6M-12M MACE, St, MI, CD, TLR, ISR | 6M  LLL 0.10﻿±0.32 vs 0.01 ±0.33 (0.08 non inferiority margin 0.35) | 12M  0 vs 6% (0.493) | 12M  0 vs 0 (1) |
| **Nishiyama et al**  **﻿10.1016/j.ijcard.2016.07.156** | 1:1 RCT  Open-label  observational  Single-center  Autonomous QCA | Sequent please PCB | EES | 60 | De novo lesions with good preparation (IVUS based) | ACS, ISR | Non-slip (NSE) recommended | Length <25mm | 8M TLR and LLL | - | 8M  LLL 0.25±0.25 vs 0.37±0.40 (0.185) | - | 8M  0 vs 6.1 (0.193) |
| **MYOCARDIAL INFARCTION** | | | | | | | | | | | | | |
| **REVELATION,2019**  **﻿10.1016/j.jcin.2019.04.016** | 1:1 RCT  Open-label non-inferiority  Single-center  Corelab  CEC | Pantera Lux | Orsiro | 120 | STEMI referred to PCI  Good predilatation result (DS<50%) | Previous Mi, recent stent implantation, controindication to DAPT, cardiogenic shock | . | Any | 9M FFR | 9M LLL, 9M MACE, St, bleeding | 9M  FFR 0.92﻿± 0.05 vs 0.91±0.06 (0.027) | 9M  0 vs 0 (1) | 9M  3 vs 2 (1) |
| **DEBAMI, 2012**  **﻿10.1016/j.jacc.2012.02.027** | 1:1:1 RCT  Open-label non-inferiority  Two-center  Corelab  CEC | DIOR+BMS | BMS vs DES | 150 | STEMI referred to PCI  Good predilatation result (DS<50%) | Controindication to DAPT, LE<12M,3VD, LM, DM+typeC lesion | 60 | 2.5-4  Length<25 | 6M LLL | 6M ISR, MACE  6M OCT and endothelial function | 6M  LLL DCB+BMS 0.74±0.57 vs BMS 0.64±0.56 vs DES 0.21±0.32 (<0.01)  ISR 26.2 vs 28.6 vs 4.7 (0.01) | 6M  23.5 vs 20 vs 4 (0.02) | - |
| **Gobic et al, 2017**  **﻿10.1016/j.amjms.2017.07.005** | Single center prospective  1:1 RCT  Autonomous QCA | Sequent Please | SES | 75 | STEMI with de novo lesion | Allergies, stroke<6M, GFR<30, LE<12M, ISR, PCI/CABG<6M, tortuosity, | - | 2.5-4 | 6M MACE | 6M LLL | 6M  LLL-0.09±0.09 vs 0.10±0.19 (0.05) | 6M  0 vs 5.4 (0.29) | - |
| **PEPCAD NSTEMI, 2019**  **10.4244/EIJ-D-19-00723.** | 1:1:1 RCT  Open-label non-inferiority  Multi-center | Sequent Please and sequent please neo | BMS (56%) and DES (44%) | 210 | NSTEMI and identifiable culprit lesion | Large thrombus | 99.2%  (bailout 15%) | Any | 9M TLF | 9M MACE | - | 9M  ITT 6.7 vs 14.2 (0.11)  PP 5.9 vs 14.4 (0.056) | 9M  TLF  ITT 3.8 vs 6.6 (0.11)  PP 4.7 vs 6.3 (0.75) |
| **Hao et al, 2021**  **10.1186/s13019-021-01525-8** | 1:1 Randomized single center prospective trial  Autonomous QCA | Biotech Bingo | DES | 80 | STEMI<12H | Severe calcification, history of bleeding, intracranial disease, cardiogenic shock  ISR, stent<6M, contraindication to DAPT | - | 2.5-4 | 12M LLL | 12M MACE | 12M  LLL -0.11±0.45 vs 0.13±0.3 (<0.05) | 12M  11 vs 12% (ns) | - |
| **PEBSI, 2017**  **10.4244/EIJ-D-16-00128** | 1:1 RCT  Open-label non-inferiority  Multi-center  Corelab  CEC | BMS + Pantera Lux | BMS | 223 | STEMI | Cardiogenic shock, LE<12M  LM, bifurcation with SB>2.5, St, more than one stenosis in same artery, referred to CABG within 30D | 18% | 2.5-4mm  Length<30mm | 9M LLL | 9M ISR and struts coverage (OCT), 9M MACE | 9M  LLL 0.31 vs 0.80 (0.001)  ISR 2.2 vs 29.8 (0.001) | 9M  3.6 vs 12.5 (0.016) | 9M  1.8 vs 7.1 (0.06) |
| **Besic, 2014**  **﻿10.1016/j.jjcc.2014.05.007** | Single center prospective  1:1 RCT  Autonomous QCA | Elutax or Sequent Please + BMS | BMS | 85 | NSTEMI/UA | STEMI, cardiogenic shock, major bleeding<2W, haemorrhagic diathesis, contraindication to DAPT  ISR, LM | 39% | Any | 6M LLL and ISR | 6M TLR, St, ACS | 6M  LLL 0.22 vs 0.68 (0.002)  ISR 17 vs 22 (0.593) | 6M  24 vs 29 (0.835) | 6M  19.5 vs 22.7 (0.770) |
| **HIGH BLEEDING RISK** | | | | | | | | | | | | | |
| **DEBUT RCT, 2019**  **﻿10.1016/ S0140-6736(19)31126-2 See** | 1:1 RCT  Open-label non-inferiority  Multi-center  Corelab  CEC | Sequent Please | BMS | 208 | De novo ischemic with at least one risk factor for bleeding | STEMI, cardiogenic shock, bifurcation (2 stents), ISR, LE<1Y, CTO, LM, suboptimal predilatation | 100 | 2.5-4 | 9M MACE | 9M TLR, procedural success  36M TLR, MACE | - | 1 vs 14% (0.00034 superiority) | 0 vs 6% (0.015 superiority) |
| **Shin et al, 2019**  **﻿10.1097/MCA.0000000000000755** | 1:1 RCT  Open-label non-inferiority  Autonomous QCA | Sequent Please | BMS | 40 | De novo HBR with FFR>0.80 post-predilatation | Cardiogenic shock, LVEF<35%, STEMI<72H, LM, 3VD, CTO, grafts, LE<12M | 100 | >2.8 | 9M LLL | 9M functional restenosis  12 M cardiac death, MI, lesion thrombosis, TLR | 9M  LLL 0.2﻿±0.3 vs 1.2﻿±0.8 (<0.001)  9M functional restenosis 0 vs 25% (0.049) | 0 vs 0 | 0 vs 0 |

ACS, acute coronary syndrome; CABG, coronary artery bypass graft; NSTEMI, non-ST segment elevation myocardial infarction; remaining as for Table 2.

**Supplementary Table 5. Image the vulnerable plaque**

|  | **TCFA** | **VULNERABLE PLAQUE** |
| --- | --- | --- |
| **OCT** | Large necrotic core, covered by a thin rim of fibrous tissue (typically ≤75 μm) | - MLA <3.5 mm2, - Fibrous cap thickness of <75 µm - Lipid arc of >180° - Macrophages |
| **VH-IVUS** | >10% confluent necrotic core at the lumen on three consecutive frames | - MLA ≤4 mm2 - Plaque burden ≥70%, - TCFA phenotype |
| **IVUS-NIRS** | Lipid rich plaque: max lipid core burden index (LCBI) within any 4 mm (LCBI_4mm_) ≥325  LCBI is the ﻿number of pixels with probability of lipid >0.6 divided by all analysable pixels multiplied by 1000 | - Plaque burden ≥70% - Lipid-rich plaque (maxLCBI_4mm_≥325) |
| **CCTA** | - | - Positive remodeling - Low-attenuation plaque - Spotty calcification - Napkin-ring sign |

CCTA, Coronary computed tomography angiography; IVUS, intravascular ultrasound; MLA, minimal lumen area; NIRS, near-infrared spectroscopy; OCT, optical coherence tomography; TCFA, thin-cap fibroatheroma; VH, virtual histology

**Supplementary Table 6. Clinical trials evaluating the performance of DCBs bifurcation lesions**

| **Study name** | **Design** | **DCB** | **Comparator** | **n** | **Inclusion** | **Exclusion** | **Pre-dilatation** | **Bailout stenting** | **Reference vessel** | **Primary endpoint** | **Secondary endpoints** | **Angiographic follow-up (p value)** | **MACE (p value)** | **TLR (p value)** |
| --- | --- | --- | --- | --- | --- | --- | --- | --- | --- | --- | --- | --- | --- | --- |
| **DEBIUT, 2012**  **﻿10.1002/ccd.23499** | 1:1:1 RCT  Open-label superiority  Multi-center  Corelab  CEC  PROVISIONAL T   - MV DCB+BMS and SB DCB - MV BMS +POBA SB - MV DES + POBA SB | Dior | PES | 117 | CCS, UA, ischemia  De novo (DS 50-100) | LVEF<30, MI<72H, LM, previous PCI in TV, severe calcification, bleeding diathesis, TIA/Stroke<3M, Major surgery planned<9M, LE<12M, contraindication to DAPT | DCB predilatation | 10 vs 5 vs 5 (0.68) | >2.5 in MV  >2 in SB | 6M LLL | 6M ISR  12M MACE | 6M  MV LLL  0.58±0.65 vs 0.60±0.65 vs 0.13±0.45 (0.87)  SB LLL  0.19±0.55 vs 0.21±0.57±0.11±0.43 (0.92)  Restenosis 24 vs 28vs15 (0.79) | 12M  20vs29vs17 (0.32) | 12M  20 vs 27 vs 15 (NS) |
| **BABILON, 2014**  **10.4244/EIJV10I1A10** | 1:1 RCT  Open-label non-inferiority  Multi-center  PROVISIONAL T   - MV DCB+ BMS and SB DCB - MV DES + POBA SB | Sequent please | EES | 108 | CCS, US, ischemia  De novo (DS 50-100) | STEMI<48H, LM, ISR, allergies, bleeding diathesis, crea>2, LVEF<35%, cardiogenic shock, stroke<6M, contraindication to DES, LE<12M | DCB predilatation in MV and SB | 7.8 vs 8.9 (1.0) | >3 in MV  >2 in SV | 6M LLL | 9M MACE | 9M  MV LLL  0.31±0.48 vs 0.16±0.38 (0.15)  SB LLL  -0.04±0.76 vs 0.03±0.51 (0.98)  Restenosis MV/SB 13/6 vs 1.8/3.6 (0.027/0.67) | 9M  17.3 vs 7.1 (0.11) | 9M  15.4 vs 3.6 (0.045) |
| **PEPCAD V, 2011** | Observational, dual-center prospective, single-arm  Pilot study  Corelab  CEC  MV DCB+BMS and SB DCB | Sequent please | - | 28 | CCS or UA or ischemia  De novo (DS>70 or >50 with ischemia) | MI, NYHA IV, cardiogenic shock, stroke, GFR<30 | DCB predilatation in MV and SB | 14.3 | MV 2.5-3.8  SB 2-3.5  LL<20 | 9M LLL | 9M TLR | 9M  MV LLL  0.38±0.46  SB LLL  0.21±0.48  Restenosis MV/SB 3.8/7.7% | 9M  10.7% | 9M  3.8% |
| **HERRADOR 2013** | Comparative observational non randomized cohort single center  Autonomous QCA  PROVISIONAL T   - MV DES and SB DCB - MV DES | Sequent Please | Taxus | 100 | DS>50% in MV and SB | Cardiogenic shock, akinetic territory, LVEF<30%, contraindication to DAPT | DCB predilatation in SB | ? | >2.5 in MV and SB  Sb LLL <10 | 12M LLL | 12M MACE | 12M  MV LLL  0.49±0.6 vs 0.62±0.7 (0.39)  SB LLL  0.09±0.4 vs 0.4±0.5 (0.01)  Restenosis MV/SB 12/7 vs 18/20 (0.44/0.08) | 12M  11 vs 24 (0.76) | 12M  12 vs 20 (0.16) |
| **DEBSIDE, 2015** | Multicenter observational  Corelab CEC  PROVISIONAL T   - SB predilatation followed by MV DES and KB and final SB DCB | Danubio | Nile PAX | 52 | CCS or UA or ischemia | PCI to TV<6M, LM, MI<72H | DCB final dilatation in SB (after provisional) | excluded | MV2.5—3.5  SB 2-3  SB LL<6 | 6M LLL | 6M ISR  6M MACE | 6M  MV LLL  0.54±0.6  SB LLL  -0.04±0.34  Restenosis MV/SB 0/0% | 6M  10% | 6M  8% |
| **BIOLUX-1, 2015**  **﻿10.1016/j.carrev.2015.07.009** | ﻿Prospective, multi-center, single arm pilot study  DCB dilatation to SB and DES to MV, final KBI with POBA | Pantera lux | - | 35 | DS>50% | Graft, significant stenosis prox or distal, tortuousity, aorto-ostial, LM, severe calcification, thrombus | DCB for SB treatment after pre-dilatation | 11.4% | MV 2-4 | 9M LLL | 12M MACE | 9M  MV LLL  0.28±0.59  SB LLL  0.10±0.43  Restenosis MV/SB 0/0% | 12M  5.7% | 12M  2.9% |
| **SARPEDON, 2015**  **﻿10.1016/j.ijcard.2015.04.002** | Single center, prospective observational cohort study  Autonomous QCA  DCB in SB after Provisional DES and KBI | Pantera Lux | - | 58 | De novo DS>50% in MV or SB | LVEF<30, heavy calcification, contraindication to DAPT, LE<2Y | - | Excluded | MV>2.25  SB>2 | 6M LLL | 12M MACE, TVR | 6M  MV LLL  0.21±0.35  SB LLL  0.09±0.21  Restenosis MV/SB 4/6% | 12M  19% | 12M  5.2% |
| **Schulz, 2014**  **﻿10.1007/s00392-014-0671-9** | Prospective single center observational study  DCB in SB, MB, SB/MB | Sequent please | - | 39 | De novo after appropriate pre-dilatation with 0.8/1:1 balloon (DS<30%MV, <75%SB) | None | 100 | 12.8 | Any | 4M ISR | 4M MACE | 4M  Restenosis MV/SB 6.7/3.3 | 4M  7.7 | 4M  7.7 |
| **PEPCAD BIF**  **10.1007/s00392-015-0957-6** | Prospective, multicenter, 1:1 RCT  Provisional DES  After successful pre-dilatation (recoil<30%, Diss<C) randomization to  - SB DCB  - POBA DCB | Sequent please | POBA | 64 | De novo Medina 0,0,1 or 0,1,1  CCS or UA, ischemia | MI<48H, NYHA IV, SVHD, LE<12M  LM, CTO | 100 | 0 | SB >2-3.5 and lesion<10mm | 9M LLL | 9M ISR | 9M  LLL 0.13±0.31 vs 0.51±0.66 (0.045)  9M ISR 6 vs 26% (0.045) | - | - |
| **Okutsu, 2022**  **10.1007/s00380-021-02000-z** | Observational, single center  Direct coronary atherectomy + DCB  LM 59%  Autonomous QCA/IVUS/OCT | Sequent please | - | 25 | De novo True bif of proximal Left coronary | STEMI, CTO, shock, severe calcification, diffuse disease | - | 5% | MV>3, SB>2 (and relevant) | Number of stents used  In-hospital MACE | 3M MACE  3M LLL | 3M  ﻿0.2 ± 0.6 mm | 0 | 0 |
| **DCA/DCB registry, 2020**  **10.1002/ccd.29185.** | Multicenter retrospective registry  Direct coronary atherectomy + DCB in bifurcation  LM 81% | Sequent please | - | 129 | Major bifurcation (SB>2mm) suitable for DCA delivery | LVEF<30, grafts, severe tortuosity, calcification, ISR, CTO, thrombus, MI<7D, dissection D-F | . | ? | SB>2 | 12M TVF | Procedure-related MACE  12M ISR  12M MACE, TLR, TVR | 6-15M  LLL ﻿0.29 ± 0.51 mm  12M ISR 2.3% | 12M  TVF  10.9% | 12M  3.1% |
| **BEYOND, 2020**  **10.1097/CM9.0000000000000743** | Prospective, multicenter RCT  DCB vs POBA in non-LM bifurcations after provisional with DES in MV (1:1 Randomization after pre-dilatation) | Bingo PEB | POBA | 222 | CCS or UA, ischemia, old MI  De novo bifurcation with SB DS>70, <50 after pre-dilatation | LM  ISR, LVEF<35%, LE<12M | 100% | 0 | SB 1.25-5, lesion length<40mm | 9M DS | TLR, TVR, TVF, MACE | 9M  DS 28.7±18.7 vs 40±19 (0.001 superiority) | 9M  0.9 vs 3.7 (0.16)  MI 0vs 0.9 (0.49) | - |
| **Liu et al, 2022**  **10.1155/2022/8250057** | Retrospective single center observational  LM with DES to MV and DCB to SB | Bingo | 2 stent strategy | 100 | True LM bifurcation | CABG, severe calcification, CTO, MI acute | 98 | - | - | 6M LLL | MACE, TLR | 6M  SB LLL  -0.17 vs 0.43 (0.001)  ISR 7 vs 30 (0.093) | 6M  No difference | 6M  6 vs 12 (0.485) |

MV, main vessel; SB, side-branch; QCA, quantitative coronary analysis; IVUS, intravascular ultrasound; OCT, optical coherence tomography; KBI, kissing balloon inflation. Others as in Table 2 and 5
